# Associations between retinal arteriolar and venular calibre with the prevalence of impaired fasting glucose and diabetes mellitus: A cross-sectional study

**DOI:** 10.1371/journal.pone.0189627

**Published:** 2018-05-03

**Authors:** Kevin Phan, Paul Mitchell, Gerald Liew, Adam J. Plant, Sarah B. Wang, Aravinda Thiagalingam, George Burlutsky, Bamini Gopinath

**Affiliations:** 1 Centre for Vision Research, Department of Ophthalmology and Westmead Millennium Institute, University of Sydney, Sydney, New South Wales, Australia; 2 Centre for Heart Research, Westmead Millennium Institute, University of Sydney, NSW, Australia; Shanghai Institute of Hypertension, CHINA

## Abstract

**Background:**

This study aims to explore retinal vessel calibre in individuals at risk of coronary artery disease (CAD), diagnosed with impaired fasting glucose (IFG) or diabetes mellitus (DM), and whether indices of CAD extent and severity modifies these associations with DM.

**Methods:**

A cross-sectional study was undertaken of 1680 patients presenting to Westmead Hospital (Sydney, Australia) for evaluation of potential CAD. Baseline digital retinal photographs, cardiovascular risk factor measurements, fasting blood tests and self-reported diabetes by patient questionnaire was recorded. Extent and severity of CAD was assessed using Extent and Gensini scores from angiography findings, respectively. Multivariate analysis including age and hypertension was undertaken to assess the association between retinal vessel calibre and IFG or DM.

**Results:**

A total of 748 patients were included; 96 (12.8%) and 189 (25.3%), respectively, had IFG or DM (together termed ‘hyperglycaemia’). No consistent association between hyperglycaemia and retinal arteriolar calibre was apparent. Wider retinal venular calibre (second and third tertile) carried a significantly higher odds of DM in men only (multivariable-adjusted OR 2.447, p = 0.005; and OR 2.76, p = 0.002; respectively). No equivalent association was apparent in women. This association was marginally significant (p = 0.08) in patients with CAD Extent scores below the median (i.e. less diffuse CAD). Retinal vessel calibre was not associated with impaired fasting glucose.

**Conclusions:**

This study reports a significant association between retinal venular widening and diabetes mellitus in men. This association was marginally stronger among participants with less diffuse CAD.

## Introduction

Diabetes mellitus (DM) is a major cause of morbidity and its microvascular complication, diabetic retinopathy, which is a leading cause of blindness in working aged adults [1]. Whilst classic retinal vascular changes in diabetic retinopathy are well-described [2], a better understanding of early pathophysiological alterations and correlations is crucial in improving treatment and prevention [3].

The retinal vessels uniquely allow non-invasive visualisation of microvascular health. Previous attempts to quantify retinal vessel calibre by ophthalmoscopy were difficult [4] but recent advances in digital retinal photography now enable accurate and reproducible measurements [5–8]. Multiple studies have since demonstrated associations between retinal vessel calibre and cardiovascular risk factors/events including hypertension [9], metabolic syndrome [10], obesity [11], coronary artery disease [12] and stroke [13]. Of particular interest is the relationship between retinal vessel calibre and DM or impaired fasting glucose (IFG) reported in various cross-sectional studies [11, 14–17]. Several large prospective population studies have further suggested retinal vascular changes precede, and may even be predictive of, incident diabetes mellitus and results implicate a mix of both retinal arteriolar and venular calibre changes [18–23]. In known persons with diabetes, retinal vessel calibres have also been linked to the development of disease complications including retinopathy [14, 15] and nephropathy [14, 24, 25].

In this study, we aimed to explore retinal vessel calibre in a patient population with diagnosed with either normoglycemia, IFG or DM, and examine these associations stratified by the severity and extent of coronary artery disease as part of the Australian Heart Eye Study. To our knowledge, the inter-relationships between retinal vessel calibre, glycaemic status and coronary artery disease have not previously been described.

## Methods

This project was approved by the Western Sydney Local Health Network Human Research Ethics Committee. Written consent was obtained from all involved patients whom were informed of the objectives, risks, costs and benefits of the study.

### Participants

The Australian Heart and Eye Study (AHES) is a cross-sectional study encompassing 1680 patients who presented to Westmead Hospital (Sydney, Australia) for diagnosis of coronary artery disease by coronary angiogram (S1 and S2 Files). All eligible consecutive patients between June 2009 and January 2012 were included. No absolute contraindications to coronary angiography were enforced and the procedure was performed where the risk-benefit ratio was favourable. Relative contraindications included contrast allergy and renal impairment. Most patients were recruited post-angiogram and investigations/data were collected as part of routine care.

Data collected included: detailed medical history, visual acuity, biochemistry, angiographic and peripheral arteriolar wave form analysis, pulse wave form, ankle-brachial index, peripheral and invasive blood pressure measurements, transthoracic echocardiogram, electrocardiogram, blood analysis and digital retinal photography. Patients were excluded if information regarding retinal vessel calibre, diabetic retinopathy or age-related macular degeneration status were incomplete.

### Medical history

A 252-item questionnaire (S3 File) was administered encompassing medical history and cardiovascular risk factors including family history. Data were collected and collated by two study personnel creating a single entry. Questionnaire categories included: cardiac rhythm, angina history, previous myocardial infarction, previous angiograms, previous cardiac intervention (open surgical bypass or percutaneous coronary stenting), hypertension (defined as participants with a formal diagnosis of hypertension or using anti-hypertensive agents), hypercholestrolemia, diabetes mellitus (defined as patients with formal diagnosis or using hypoglycemic agents or insulin) or impaired fasting glucose state, chronic medical conditions, current medication, smoking status, alcohol consumption, previous stroke/transient ischemic attack.

### Retinal vessel calibre assessment

Digital retinal photography was used to assess retinal vessel calibre. A pre-calibrated Canon 60^o^ fundus camera (Model CF-60DSi, Canon Inc., Tokyo, Japan) with an attached digital camera (Model 1DSmklll, Canon Inc., Tokyo, Japan) was utilised to capture dilated images of the optic disc and macula bilaterally. Retinal vessel calibre measurements from the right eye of each participant were used preferentially unless ungradable. One masked study personnel measured retinal vessel caliber using a computer-assisted program (IVAN, University of Wisconsin, Madison) as previously described [5, 12] and these studies reported high reproducibility with this methodology. For the present study intra-grader reliability of this method was high with quadratic weighted kappa values of 0.85 (CRAE) and 0.90 (CRVE). The diameter of all retinal vessels coursing through a pre-selected retinal region was measured. Values were combined with the Parr-Hubbard formula as modified by Knudtson, Lee [26] to obtain an average representing the central retinal arteriolar/venular equivalent (CRAE, CRVE).

### Assessment of covariates

Peripheral blood pressure was measured using the Intellisense^TM^ OMRON digital automatic blood pressure monitor (Model HEM-907; OMRON Healthcare, Singapore) as a single measurement in the right arm in supine position. Invasive blood pressure measurements were achieved via a fluid filled catheter in the central aorta attached to the Mac-Lab hemodynamic system (GE Healthcare Milwaukee, WI). Diabetic status was determined from self-report, reported use of diabetes medications and blood glucose levels. Where the status was unknown or undetermined the patient was excluded from the study. Data collected from patient records included: renal function, full blood count, cardiac enzymes (creatinine kinase, troponin T), fasting blood glucose, HbA1c, fasting lipids and thyroid function tests.

### Assessment of coronary artery disease

Routine diagnostic coronary angiography was performed after six hours fasting via either the femoral or radial artery using a catheter of known dimension (5Fr to 7Fr). Selective coronary injections of Ultravist (Schering) were filmed in standard projections on a Siemens Bi-Plane radiographic unit (Siemens Healthcare, Germany).

All angiograms were analyzed offline by a trained cardiologist masked to the results of the adjunctive investigations and retinal grading. The coronary artery segments were defined using the Syntax system, which divides the arterial tree into 16 segments, based on the modified American Heart Association (AHA) classification[27]. For each segment, the severity of obstruction was documented using several grades: normal, 1–25%, 25–50%, 50–74%, 75–99% and 100% (occluded). Each lesion that was visually scored as greater than 50% luminal obstruction in a vessel that was ≥1.5mm diameter was further analyzed using quantitative coronary analysis (QCA). QCA was performed using validated computerized edge-detection software (QCAPLUS, Sanders data Systems, Palo Alto, California, USA).

Coronary angiograms were scored according to two methods to document both the severity and extent of CAD:

(i) Gensini score (severity score): This has been described previously[28]. Briefly, the coronary arterial tree was divided into segments with multiplying factors according to the functional importance of any given segment (5 for the left main trunk to 0.5 for the most distal segments) and the percentage reduction in luminal diameter of each narrowing was assigned a score (0, 1, 2, 4, 8, 16 or 32), according to the degree of stenosis. The sum of the scores of all segments provides the Gensini score, which places emphasis on the severity of the disease[29].
(ii) Extent score: The extent score was proposed by Sullivan et.al to define the proportion of the coronary arterial tree with angiographically detectable coronary atheroma[30]. The proportion of each vessel involved by atheroma, identified by lumen irregularity, was multiplied by a factor for each vessel, which is related to the length of that vessel. The scores for each vessel were added to give a total score out of 100. This percentage represents the proportion of the coronary intimal surface area containing coronary atheroma[29].

### Statistical analysis

Statistical analysis was performed using SAS (version 9.2, SAS Institute Inc., Cary, North Carolina, USA) and statistical significance was defined as P < 0.05. Multivariate logical regression analysis involved initially adjusting for age and sex, and then further adjusted for hypertension, BMI, smoking, diabetes history, cholesterol level and prior statin use. Confounders were selected based on previous studies reporting significant association with retinal vessel caliber [9–12].

## Results

**Fig 1:**
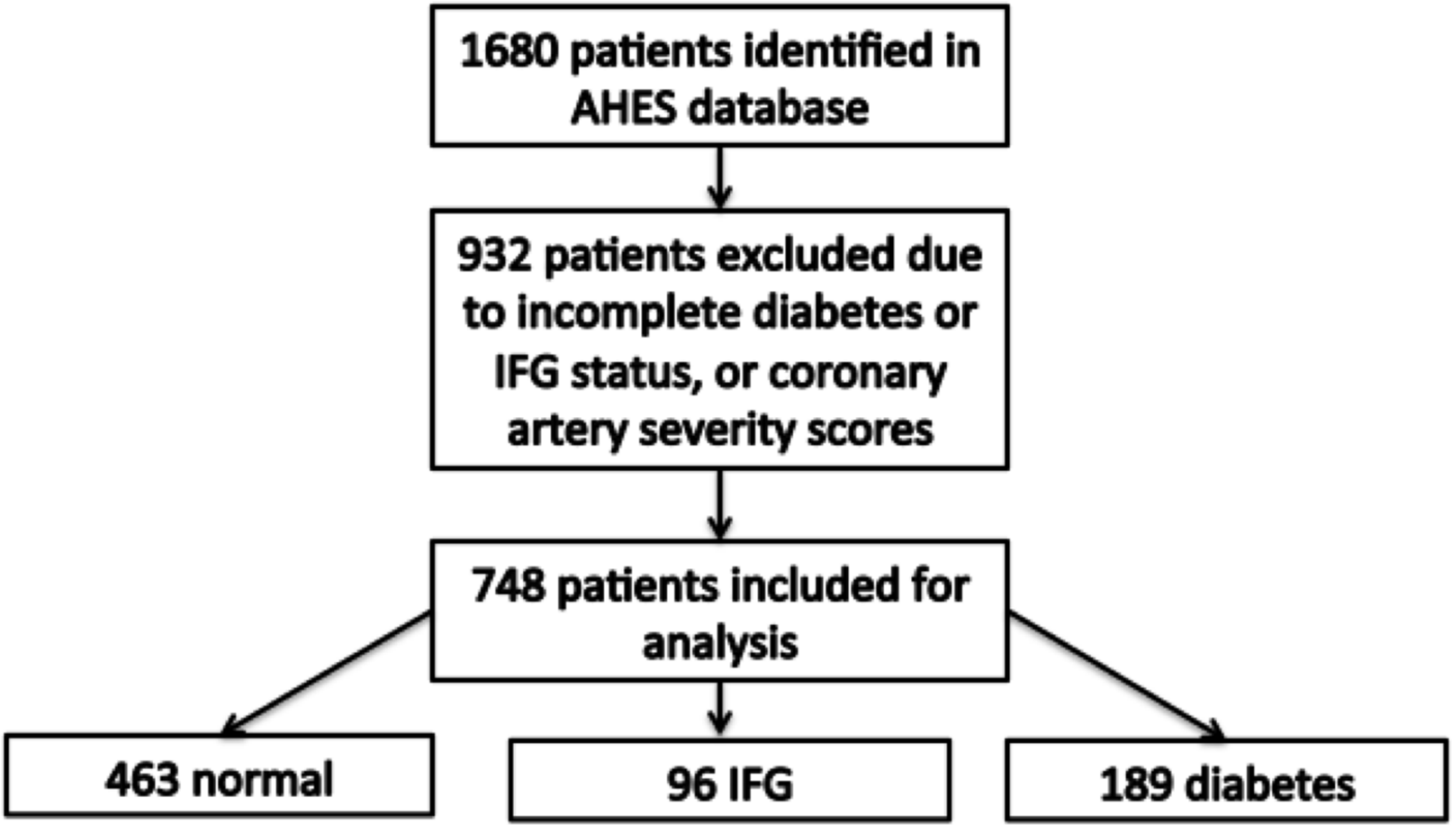
Flow chart demonstrating identification of cases for analysis in the present study.

A total of 748 patients were included in the cross-sectional analysis of which 96 (12.8%) and 189 (25.3%) IFG and DM, respectively (Fig 1). Baseline characteristics are shown in Table 1. The baseline characteristics of the 748 patients included in this study were compared with 932 patients of the AHES study who were not included for analysis (Table A of S4 File). Significant differences were found in the proportion of males, smokers, history of hypertension, alcohol consumption, statins use, and retinal vessel calibre (Table A of S4 File). For the present study, age and gender did not differ significantly between the groups but patients with IFG and DM compared to healthy participants had greater BMI (30.56±5.67 vs. 30.88±6.33 vs 28.85±5.51, p<0.0001). Blood pressure, other cardiovascular risk factors and history of cardiac events did not differ significantly between the groups (Table 1).

**Table 1:** Demographic and clinical characteristics of participants.

Characteristics	Normal (n = 463)	Impaired fasting glucose (n = 96)	Diabetes (n = 189)	P-value
Age	60.32±11.92	60.71±10.49	61.68±11.44	0.40
Male	363 (78.4%)	81 (84.4%)	141 (74.6%)	0.17
BMI	28.85±5.51	30.56±5.67	30.88±6.33	<0.001
Systolic Blood pressure (mmHg)	124.0±18.67	125.78±18.85	123.72±17.77	0.65
Diastolic pressure (mmHg)	72.53±12.31	73.30±12.36	72.19±12.88	0.78
Mean arterial pressure (mmHg)	89.68±12.92	90.75±13.39	89.34±12.94	0.68
Smoking	163 (35.2%)	25 (26.0%)	62 (32.8%)	0.25
History of hypertension	277 (59.8%)	58 (60.4%)	130 (68.8%)	0.11
Alcohol consumption (rarely+often)	349 (75.4%)	65 (67.7%)	131 (69.3%)	0.46
HDL	1.03±0.30	1.01±0.25	1.01±0.30	0.73
Cholesterol	4.65±1.09	4.68±1.11	4.50±1.16	0.30
Statin therapy	57 (35.0%)	14 (14.6%)	37 (19.6%)	0.07
History of AMI	114 (24.6%)	25 (26.0%)	53 (28.0%)	0.61

BMI, body mass index; HDL, high density lipoproteins; AMI, acute myocardial infarctions

Initial adjustments for age revealed that patients in the first or second tertile of retinal arteriolar calibre did not have significantly different odds compared to patients in the third tertile for the presence of DM or IFG. Interaction was calculated between sex and retinal arteriolar calibre and was determined to be significant (0.02) and therefore we decided to stratify our analyses according to gender. Initial adjustments for age revealed that men in the second tertile of retinal arteriolar calibre had significantly higher odds (OR 1.75, p = 0.03) of having DM compared to the third tertile (reference, widest calibre) group (Table 2). This association did not persist after multivariate adjustment. Conversely, females in the second tertile of retinal arteriolar calibre had a significantly lower odds (OR 0.30, p = 0.03) of having DM compared to the third tertile group, after multivariate adjustment.

**Table 2:** Cross-sectional association between adjusted retinal vessel calibre and diabetes.

Retinal vessel calibre	All				Men (n = 585)				Women (n = 163)			
	Age-adjusted OR	P-value	Multivariable* adjusted OR	P-value	Age-adjusted OR	P-value	Multivariable* adjusted OR	P-value	Age-adjusted OR	P-value	Multivariable* adjusted OR	P-value
Adjusted retinal arteriolar calibre (μm)												
1^st^ tertile	1.26 (0.74–2.17)	0.40	1.13 (0.58–2.20)	0.71	1.43 (0.87–2.33)	0.16	1.23 (0.69–2.19)	0.49	0.77 (0.33–1.81)	0.55	0.65 (0.21–1.95)	0.44
2^nd^ tertile	1.16 (0.66–2.02)	0.61	0.89 (0.44–1.78)	0.73	1.74 (1.06–2.85)	0.03	1.15 (0.63–2.08)	0.65	0.44 (0.19–1.02)	0.055	0.30 (0.10–0.89)	0.03
3^rd^ tertile (reference)	1.0	-	1.0	-	1.0	-	1.0	-	1.0	-	1.0	-
Adjusted retinal venular calibre (μm)												
1^st^ tertile (reference)	1.0	-	1.0	-	1.0 (reference)	-	1.0 (reference)	-	1.0 (reference)	-	1.0 (reference)	-
2^nd^ tertile	1.84 (1.19–2.855)	0.007	2.21 (1.30–3.76)	0.004	1.85 (1.10–3.11)	0.02	2.45 (1.31–4.5 9)	0.005	1.91 (0.82–4.42)	0.13	2.29 (0.73–7.17)	0.16
3^rd^ tertile	1.94 (1.25–3.02)	0.003	1.98 (1.16–3.39)	0.01	2.45 (1.45–4.10)	0.0007	2.76 (1.46–5.21)	0.001	0.94 (0.39–2.30)	0.89	0.73 (0.23–2.26)	0.58

*Multivariate analysis: age, BMI, alcohol, smoker, hypertension, history of diabetes, cholesterol level, HDL level, prior statin use; the retinal arteriolar model was adjusted for retinal venular calibre; the retinal venular model was adjusted for retinal arteriolar calibre.

For the whole cohort, the second and third tertiles of adjusted retinal venular calibres were associated with significantly higher odds of DM compared to the first tertile. Initial adjustments for age revealed that men in the second and third tertile of retinal venular calibre had significantly higher odds (OR 1.85, p = 0.02; OR 2.45, p = 0.001, respectively) of having DM compared to the first tertile (reference, narrowest calibre) group (Table 2). This association persisted after multivariate adjustment (OR 2.45, p = 0.005; OR 2.76, p = 0.002; for second and third tertiles, respectively). Conversely, there were no associations between retinal venular calibre and the odds of DM in women (Table 2). There were no significant associations observed between retinal arteriolar or venular calibre and the odds of IFG (Table B of S4 File) in men or women.

The association between retinal venular calibre and diabetes was stratified according to Gensini and Extent scores for CAD by tertiles (Table 3). The only significant association found was between patients with Gensini scores in the first tertile with diabetes, however this was attenuated after multivariate adjustment.

**Table 3:** Relationship between retinal venule calibre and diabetes, stratified by Gemini / Extent score.

	Adjusted Retinal venular calibre per SD increase	
	Age-sex adjusted OR (95% CI)	Multivariable* adjusted OR (95% CI)
By Gensini score		
1^st^ tertile	1.56 (1.04–2.34)	1.11 (0.63–1.93)
2^nd^ tertile	1.17 (0.28–1.65)	1.19 (0.79–1.80)
3^rd^ tertile	1.02 (0.75–1.39)	1.02 (0.70–1.49)
By Extent score		
1^st^ tertile	1.57 (0.96–2.58)	1.34 (0.76–2.35)
2^nd^ tertile	1.42 (0.95–2.10)	1.28 (0.76–2.15)
3^rd^ tertile	1.01 (0.73–1.39)	0.96 (0.64–1.45)

*Multivariate analysis: age, sex, BMI, alcohol, smoker, hypertension, history of diabetes, cholesterol level, HDL level, prior statin use; the retinal arteriolar model was adjusted for retinal venular calibre; the retinal venular model was adjusted for retinal arteriolar calibre.

Supplementary analysis involved stratifying patients according to Gemini and Extent score, that is above and below median scores (i.e. reduced extent or severity of CAD). Participants below the median Extent score had 1.67 increased odds of having DM with each SD increase in adjusted venular calibre (p = 0.008). This relationship was however attenuated after multivariate-adjustment (p = 0.08) (Table C of S4 File).

## Discussion

In this cross-sectional population study of 748 patients we investigated the independent relationship between retinal arteriolar and venular calibre and IFG and DM. After multivariate analysis, wider retinal venular calibre was associated with increased odds of DM in men but not women. The association was marginally more significant in participants with less diffuse CAD.

### Retinal arteriolar calibre in IFG/DM

A number of large studies have reported mixed results when exploring the relationship between retinal arteriolar calibre and hyperglycemia. Cross-sectional results reporting positive association include the Australian Diabetes Obesity and Lifestyle study [15], the Multi-Ethnic Study of Atherosclerosis (MESA) study [16], where significance was sustained only in the white population, and a study by Jeganathan, Sabanayagam (17), where the relationship was particularly strong in subjects of Indian background. In agreement a prospective study in the Beaver Dam population reported an association between larger arteriolar calibre and 15 year incident diabetes [31]. Contrastingly, Wong, Shankar [23] and Nguyen, Wang [21] reported retinal arteriolar narrowing, rather than widening, to be associated with incident diabetes at 10 year follow-up in the Beaver Dam and at 5 year follow-up of the AusDiab Study, respectively.

A recent meta-analysis by Sabanayagam, Lye [32] pooling 5 population-based prospective studies confirmed no association between retinal arteriolar calibre and diabetes or impaired fasting glucose. This is in agreement with the findings reported in this current study.

### Retinal venular calibre in IFG/DM

Our finding of a positive association between retinal venule widening and DM is consistent with that reported in the literature. Cross-sectional data from the Wisconsin Epidemiological Study of Diabetic Retinopathy (WESDR) [33], MESA [16] and a Singaporean study [17] reported a greater magnitude of retinal venular widening association with presence of DM. Prospective studies later reported this relationship may be predictive with patients having wider baseline venular calibre being more likely to develop DM, as in the Beaver Dam Eye Study [20], or impaired fasting glucose, as in the Blue Mountains Eye Study among patients aged under 70 years [19]. The Wisconsin study further showed that wider retinal venular calibre was related to retinopathy severity, duration of diabetes and higher levels of glycosylated haemoglobin [14]. The association between larger retinal venular calibre and DM was also confirmed in a meta-analysis of 5 prospective population studies [32].

The mechanism of association between retinal venular calibre and DM is currently unknown. Physiological studies have highlighted the role of insulin in increasing skeletal muscle microvascular perfusion [34]. Changes in insulin status have subsequently been shown to alter microvascular recruitment where hyperinsulinemia stimulates total blood flow [35] and with insulin resistance this mechanism is impaired [34]. Whilst studies have been largely confined to skeletal muscle, a similar phenomenon may be extrapolated to the retinal microvasculature. While the selectivity for retinal venules is unclear, wider retinal venules have previously been noted as a marker of endothelial dysfunction and inflammation [36]. Thus, it is possible the selective widening of retinal venules represents the combined effect of inflammation, insulin resistance and endothelial dysfunction. Further studies are required to elucidate the pathological mechanisms underlying this relationship.

After stratification by Extent score, our data suggest this positive association was more apparent in patients with less diffuse CAD (i.e. Extent scores below the median), thus present in the absence of macrovascular disease. To our knowledge this association between retinal venular calibre and diabetes, in the presence of less diffuse CAD, has not been previously described. The loss of statistical significance with multivariate analysis in this case may be due to small sample size and further adequately-powered studies would be helpful to explore the reproducibility and magnitude of this association.

Interestingly, we report a gender difference in the association between retinal venular calibre and DM where significance was only apparent in men. This is in-line with a meta-analysis of pooled data where the association was stronger in men [32]. Benitez-Aguirre, Craig [37] previously reported sex differences in retinal vascular geometry in pre-pubertal Type 1 diabetics where young females had wider baseline retinal venular calibre and greater propensity to develop retinopathy earlier. The influence of sex hormones on the retinal vasculature and its links with hyperglycemia has not been previously explored and may be of interest in future studies.

### Limitations

In interpreting the results of our study it is important to consider its limitations. Firstly, the cross-sectional design of our study only allows description of associations and not causal relationships. However, several large prospective studies with similar findings suggest causality [19, 20]. Secondly a number of other baseline cardiovascular risk factors, in particular hypertension [38], have been shown to affect retinal vessel calibre. Multivariate adjustment for key cardiovascular risk factors facilitated evaluation of DM as an independent factor in retina vessel calibre, however, we cannot disregard the potential influence of residual confounding on observed associations. Thirdly, our retinal vessel measurements were not cardiac-gated and variation in measurements of 2–11% in venules have been reported [39]. However randomisation of measurements is likely to minimise this effect and our methods are in line with previous large studies. Fourthly, the interobserver and intraobserver variability in the cardiac scores (Extent, Gensini) have not been calculated. However, these scores have previously been validated by dedicated studies and thus used as such [40, 41]. Another interesting observation was a large number of men in the present cross-sectional study compared to females, which is likely due to bias from the cardiovascular nature of the cohort. We cannot also completely exclude the risk of selection bias, given the cross-sectional nature of this study, and as such, the present findings require confirmation in prospective longitudinal studies.

## Conclusions

These cross-sectional data from the Australian Heart Eye Study demonstrated a significant association between retinal venular widening and diabetes mellitus. Interestingly the relationship was only significant in men and not women. No significant association was observed with retinal arteriolar calibre or with impaired fasting glucose after multivariate adjustment. These results are in agreement with recent literature and meta-analysis findings thus adds to the existing evidence indicating an association between widening of the retinal venular calibre and DM. Uniquely, our data also suggest this association is marginally stronger in patients with lower Extent scores. Prospective studies are further required to understand the utility of retinal venular calibre as a predictive marker of the development of DM and its complications.

## Supporting information

S1 File Database used for the present study. (SAS7BDAT)

S2 File Key for variables in the database used for the present study. (DOCX)

S3 File Questions used to formulate the database for the present study. (DOC)

S4 File Table A. Baseline characteristics. Table B. Cross-sectional association between adjusted retinal vessel calibre and impaired fasting glucose (IFG). Table C. Relationship between retinal venular calibre and diabetes, stratified by Gemini / Extent score, according to median.
(DOCX)
