# Supplementary material for: Associations between retinal arteriolar and venular calibre with the prevalence of impaired fasting glucose and diabetes mellitus: A cross-sectional study
Source: PLoS One. 2018 May 3;13(5):e0189627. doi: 10.1371/journal.pone.0189627 (PMC5933737; doi:10.1371/journal.pone.0189627)
Supplement: S2 File — (DOCX) [file pone.0189627.s002.docx]

| **Variables in Creation Order** | | |
| --- | --- | --- |
| **#** | **Variable** | **Descriptions** |
| **1** | IDNUM |  |
| **2** | Glucose | Fasting glucose level |
| **3** | Crae6 | Retinal arterial calibers |
| **4** | Crve6 | Retinal vein calibers |
| **5** | AdjCrae6 | Crae6 adjusted for Crve6 |
| **6** | AdjCrve6 | Crve6 adjusted for Crae6 |
| **7** | CraeSTD | Standardised Crae6 |
| **8** | CrveSTD | Standardised Crve6 |
| **9** | AdjCraeSTD | Standardised AdjCrae6 |
| **10** | AdjCrveSTD | Standardised AdjCrve6 |
| **11** | diab | 0-No, 1-Undiagnosed(glucose or medicine), 2-Previosly diagnosed |
| **12** | Diabet | 0-No, 1-Diabetis |
| **13** | Age |  |
| **14** | sex | 1-Female, 2-Men |
| **15** | BMI |  |
| **16** | AMI3 | Acute myocardial infarction: 1-Yes, 0-No |
| **17** | systbp3 | Systolic BP |
| **18** | diasbp3 | Diastolic BP |
| **19** | Chol | Fasting cholesterol |
| **20** | HDL | Fasting HDL |
| **21** | EXTENT | Extent score |
| **22** | GENSINI | Gensini score |
| **23** | statin | Using statin: 1-Yes, 0-No |
| **24** | rGensini | * |
| **25** | r2Gensini | ** |
| **26** | rExtent | * |
| **27** | r2Extent | ** |
| **28** | Alcohol | Self report using of alcohol: 1- Never, 2 - <=4 days a week, 3 - >= 5 days a week |
| **29** | Smoker2 | 1-current smoker, 0 – not smoker |
| **30** | Hypert | 0 – No, 1 – using hypertension drug or (SBP>145 or DBP>90) |
| **31** | fDiab | Famili history of diabetes: 1-Yes, 0-No |
| **32** | MAP | Mean Arterial Pressure |
| **33** | rGL | 0 - Gucose<6.1, 1 - 6.1<=Glucose<7, 2 – Glucose>=7 |
| **34** | rGL1 | If rGL in(0,1) then rGL1=rGL |
| **35** | rGL2 | If rGL in(0,2) then rGL2=rGL |

* 0 - if Gensini score (Extent score) equal 0, if not then 1,2,3 – tertiles of non 0 scores;

** 0 - if Gensini score (Extent score) equal 0, if not 1- scores<median, 2- scores >=median
