# Supplementary material for: Associations between retinal arteriolar and venular calibre with the prevalence of impaired fasting glucose and diabetes mellitus: A cross-sectional study
Source: PLoS One. 2018 May 3;13(5):e0189627. doi: 10.1371/journal.pone.0189627 (PMC5933737; doi:10.1371/journal.pone.0189627)
Supplement: S3 File — (DOC) [file pone.0189627.s003.doc]

**DATE OF INTERVIEW:** □□/□□/□□□□(dd/mm/yyyy)

**INTERVIEW CONDUCTED BY:**________________________________________________

**LOCATION OF INTERVIEW:__________________________________________________**

**START TIME:___________________**

**FINISH TIME:___________________**

# GENERAL DEMOGRAPHICS

**1. IDENTITY**

1. {idnum} Study ID number: □□□□
2. {idwmh} Westmead MRN: □□□□□□□□
3. {surname} Surname: ________________________________________________________­
4. {name} First name (s): _____________________________________________________
5. {sex} Sex: **□**1 Female **□**2 Male
6. {dob} Date of birth: □□/□□/□□□□(dd/mm/yyyy)
7. {address} Your current address? __________________________________________________

_____________________________________________________________________

1. {postcod} Postcode □□□□
2. {usuadrs} Is this your usual address (live here more than 6 months of the year)? **□**1 Y **□**2 N
3. {phno} Your phone number? ___________________________________________________
4. {othadd} Is your mailing address different to above? **□**1 Y **□**2 N **□**8 DK **□**9 Missing
5. {addoth} If **yes**, mailing address: _________________________________________________

_____________________________________________________________________

1. {postoth} Postcode □□□□
2. {marital} What is your **current marital status**?

**□**1 Never married **□**4 Divorced

**□**2 Married **□**5 Widowed

**□**3 Separated but not divorced **□**6 De-facto **□**8 DK **□**9 Missing

1. {eng} Do you speak English at home? **□**1 Yes **□**2 No
2. {lang} If **no**, which language do you most often speak at home?

If **yes**, do you speak another language at home?

____________________________________________________ | code _________

1. {ancest} What is your parents’ ancestry?

Mother: Father:

__________________ | code _________ __________________ | code _________

1. {jobstat} Are you retired or still employed?

**□**1 House duties **□**5 Unemployed

**□**2 Retired **(go to 1-19)**  **□**6 Other: ___________________________

**□**3 Employed **(go to 1-20)** **□**8 DK

**□**4 Medical disability **□**9 Missing

1. {retired} If **retired**, how old were you when you retired? __________________ years

**□**8 DK **□**9 Missing

1. {presjob} If **employed**, what is your present occupation?

_________________________________________________ | code _________

1. {mainjob} In your working life, what has been your **main job**?

_________________________________________________ | code _________

1. {othjobs} Could you list other jobs you have had (≥ 5 years)

_________________________________________________ | code _________

_________________________________________________ | code _________

_________________________________________________ | code _________

_________________________________________________ | code _________

_________________________________________________ | code _________

Could you please give us the name and address of two people we could contact to get a forwarding address for you if you move?

**Contact 1**

1. {fadnam1} Name: ______________________________________________________________
2. {fadph1} Telephone: __________________________________________________________
3. {fadd1} Address: ____________________________________________________________
4. {fadrel1} Relationship: _________________________________________________________

**Contact 2**

1. {fadnam2} Name: ______________________________________________________________
2. {fadph2} Telephone: __________________________________________________________
3. {fadd2} Address: ____________________________________________________________
4. {fadrel2} Relationship: _________________________________________________________

**General Practitioner**

1. {gpnam} Who is your **GP**? ___________________________________________________
2. {adrgp} What address is his/her surgery? _______________________________________
    __________________________________________________________________
3. {lastgp} When did you last visit your GP? __________ months ago
4. {freqgp} How often do you visit your GP? ______________ per ________________

**Ophthalmologist / optometrist**

- 1. {whoey} Who was the last person you saw for your eyes, for glasses or any eye treatment?

____________________________________________________________________

1. {wheneye} When? (specify month & year if in the last 1 year) _______/______/______ (dd/mm/yyyy)
2. {adreye} In which suburb are his/her rooms? ________________________________________
3. {optoph} Was it, **□**1 an optometrist **□**8 DK

**□**2 an eye specialist (**go to 1-43) □**9 Missing

1. {seeoph} If **not an eye specialist**, have you previously seen an eye specialist?

**□**1 Y **□**2 N **(go to 1-43)** **□**8 DK **□**9 Missing

1. {whooph} If **yes,** who was the last eye specialist you saw? ______________________________
2. {whenoph} When? (specify month & year if in the last 1 year) _______/______/______ (dd/mm/yyyy)
3. {locoph} In which suburb are his/her rooms? ________________________________________

**Cardiologist**

1. {whoh} Who was the last doctor you saw for your heart?

____________________________________________________________________

1. {whenh} When? (specify month & year if in the last 1 year) _______/______/______ (dd/mm/yyyy)
2. {adrh} In which suburb are his/her rooms? ________________________________________

**Report**

- 1. {report} If abnormalities were detected on the retinal photography, we would send a report to you, your GP and your optometrist / eye doctor.

Are you happy with this? **□**1 Yes **□**3 GP only

**□**2 Participant only **□**4 Eye doctor only

**2. HOSPITAL DETAILS**

2-1 {transp} Transport to hospital:

**□**1 ETAMI to Westmead

**□**2 Central district ambulance

**□**3 Private car

# □4 Other, specify: ___________________________ | code _________

# 2-2 {admiss} Admission type:

# □1 Booked short stay

**□**2 Inpatient at Westmead Hospital

**□**3 Inter hospital transfer, specify: ________________________

# □4 Emergency department

2-3 {medica} If not obtained, Medicare number¨🞎🞎🞎-🞎🞎🞎🞎🞎-🞎-🞎

# MEDICATIONS

I would like to ask about the tablets or other medications you are currently taking.

*Please complete as accurately as possible.*

**3. SPECIFIC MEDICATIONS**

**Aspirin**

I am now going to ask questions about your use of aspirin.

Aspirin-based drugs include: Solprin, Cardiprin, Disprin, Ecotrin, **but not Panadol or Dymadon**.

1. {aspirin} Have you taken aspirin in the last 12 months?

**□**1Y **□**2 N **(go to 3-4)** **□**8 DK **□**9 Missing

1. {aspmon} If **yes***,* how many aspirin would you typically consume per month?

Tablets per month: ______________ ***Record details of any present or past medications.***

1. {aspyr} For how many years have you been taking this amount? _________________ years

**s**

1. {stmed} Have you ever taken steroid **tablets** such as prednisone for asthma, arthritis or other conditions for more than one month?

**□**1Y **□**2 N **(go to 4-0)** **□**8 DK **□**9 Missing

If **yes**, which tablets did you take (see list)? ________________________________

***Record details of any present or past medications.***

1. {stcond} What condition were you taking steroid tablets for?

________________________________________________________│code ______

***Complete tables in conjunction with Medical and Surgical History questions.***

**4. PRESENT MEDICATIONS**

1. {currmed} Are you currently taking any medications (apart from the ones already identified)?

**□**1 Y **□**2 N **(go to** **5-0)** **□**8 DK **□**9 Missing

|  | **Name** | **Code** | **Strength (mg) per tablet** | **No. of tablets per day** | **Start**  **Year** | **Duration (months)** |
| --- | --- | --- | --- | --- | --- | --- |
| 4-1  {tab1} |  |  |  |  |  |  |
| {tab2} |  |  |  |  |  |  |
| 4-3 {tab3} |  |  |  |  |  |  |
| 4-4 {tab4} |  |  |  |  |  |  |
| 4-5 {tab5} |  |  |  |  |  |  |
| 4-6 {tab6} |  |  |  |  |  |  |
| 4-7 {tab7} |  |  |  |  |  |  |
| 4-8 {tab8} |  |  |  |  |  |  |
| 4-9 {tab9} |  |  |  |  |  |  |
| 4-10 {tab10} |  |  |  |  |  |  |
| 4-11  {tab11} |  |  |  |  |  |  |
| 4-12  {tab12} |  |  |  |  |  |  |

**Medication List**

(please attach securely)

**5. PAST MEDICATION**

1. {pastmed} Can you recall any other tablets, vitamins, drops, or other medications that you are

not taking now that you have taken for more than 3 months in the past 5 years?

**□**1 Y **□**2 N **(go to** **6-0)** **□**8 DK **□**9 Missing

|  | **Name** | **Code** | **Strength (mg)** | **No. per day** | **Start Year** | **Duration (months)** |
| --- | --- | --- | --- | --- | --- | --- |
| 5-1 {tabp1} |  |  |  |  |  |  |
| 5-2 {tabp2} |  |  |  |  |  |  |
| 5-3 {tabp3} |  |  |  |  |  |  |
| 5-4 {tabp4} |  |  |  |  |  |  |
| 5-5 {tabp5} |  |  |  |  |  |  |
| 5-6 {tabp6} |  |  |  |  |  |  |
| 5-7 {tabp7} |  |  |  |  |  |  |
| 5-8 {tabp8} |  |  |  |  |  |  |

**6. EYEDROP MEDICATIONS**

1. {eyemed} Are you currently using any eyedrops for any eye conditions?

**□**1Y **□**2 N **(go to 7-1)** **□**8 DK **□**9 Missing

|  | **Name** | **Yes No DK Missing** | **Code** | **Strength (%)** | **No. of drops per day** | **Start Year** | **Duration (months)**  **or still taking** |
| --- | --- | --- | --- | --- | --- | --- | --- |
| 6-1 {drop1} | Timolol | **□**1 **□**2 **□**8 **□**9 | N/A |  |  |  |  |
| 6-2 {drop2} | Xalatan | **□**1 **□**2 **□**8 **□**9 | N/A |  |  |  |  |
| 6-3 {drop3} | Artificial tears | Specify: |  |  |  |  |  |
| 6-4 {drop4} | Other | Specify: |  |  |  |  |  |
| 6-5 {drop5} | Other | Specify: |  |  |  |  |  |
| 6-6 {drop6} | Other | Specify: |  |  |  |  |  |

# MEDICAL AND SURGICAL HISTORY

I would like to ask some questions about your general health, to find whether this is related to eye disease.

**7. GENERAL**

1. {health} For someone of your age, how would you rate your overall health? Is it:

**□**1 Excellent **□**2 Good **□**3 Fair **□**4 Poor **□**8 DK **□**9 Missing

1. {bed} Have you spent more than a week in bed because of illness or injury in the past 3 months?

**□**1 Yes **□**2 No **□**8 DK **□**9 Missing

1. {hospad} Have you had any admissions (at least overnight) to a hospital in the last 12 months? **□**1 Yes **□**2 No **(go to 8-1)** **□**8 DK **□**9 Missing
2. {hospadm} If **yes**, number of times: _______ times **□**8 DK **□**9 Missing
3. What were you admitted for?

{hosres1} ___________________________________________________________│code _____

{hosres2} ___________________________________________________________│code _____

{hosres3} ___________________________________________________________│code _____

1. Which hospital(s)?

{hosnam1} ___________________________________________________________│code _____

{hosnam2} ___________________________________________________________│code _____

{hosnam3} ___________________________________________________________│code _____

**8. MEDICAL CONDITIONS**

**Has a doctor advised you that you have any of the following conditions:**

1. {angina} **Angina** **□**1 Yes **□**2 No **□**8 DK **□**9 Missing
   - 1. {angyr} When was it first diagnosed? _________ years ago
     2. {angecg} Was the diagnosis confirmed with an ECG? **□**1 Yes **□**2 No **□**8 DK **□**9 Missing
     3. {angdr} Name & address of doctor who made diagnosis?

___________________________________________________________________

How would you describe your chest pain?

8-1.4 {angchar} Character: **□**1 Sharp **□**4 Burning **□**7 Tightness

**□**2 Dull **□**5 Squeezing **□**8 Other, specify:

**□**3 Crushing **□**6 Heaviness ____________________

8-1.5 {angrad} Radiation: **□**1 Down arm **□**3 Up neck

**□**2 Through to back **□**4 None

8-1.6 {angas} Association with: **□**1 Sweating **□**4 Palpitations

**□**2 Dizziness **□**5 Nausea or vomiting

**□**3 Shortness of breath **□**6 Worse on inspiration **□**7 None

8-1.7 {angoth} Other symptoms: **□**1 Yes **□**2 No **□**8 DK **□**9 Missing

If **yes**, describe: _____________________________________________________

- - 1. {angtab} Do you take anti-angina tablets or sprays?

**□**1 Yes **□**2 No **□**8 DK **□**9 Missing

***Record details of any present or past medications.***

1. {mi} **Heart Attack** **□**1 Yes **□**2 No **□**8 DK **□**9 Missing

- - 1. {miyr} When was it diagnosed? _________ years ago
    2. {miecg} Was the diagnosis confirmed with an ECG? **□**1 Yes **□**2 No **□**8 DK **□**9 Missing
    3. {mibld} Was diagnosis confirmed with a blood test? **□**1 Yes **□**2 No **□**8 DK **□**9 Missing
    4. {midr} Name & address of doctor who made diagnosis?

___________________________________________________________________

- - 1. {mihsp} Were you admitted to hospital? **□**1 Yes **□**2 No **□**8 DK **□**9 Missing
    2. {mihspnm} Which hospital? ______________________________________________________
    3. {mihspt} For how long? _________ days
    4. {mirx} Treatment for your heart attack?

**□**1 Bypass (CABG) _______ years ago at ____________________ hospital

**□**2 Angioplasty (PTCA) _______ years ago at ____________________ hospital

**□**3 Thrombolytic (clot busting medication)

_______ years ago at ____________________ hospital

**□**4 Other: specify: ___________________________________________________

***Record details of any present or past medications.***

1. {arr} **Arrhythmia** **□**1 Yes **□**2 No **□**8 DK **□**9 Missing

8-3.1 {arryr} When was it diagnosed? _________ years ago

8-3.2 {arrecg} Was the diagnosis confirmed with an ECG? **□**1 Yes **□**2 No **□**8 DK **□**9 Missing

8-3.3 {arrdr} Name & address of doctor who made diagnosis?

___________________________________________________________________

8-3.4 {arrtyp} Type of arrhythmia: ___________________________________________________

8-3.5 {arrmed} Treatment of arrhythmia?

**□**1 Anti arrhythmia medications

**□**2 Pacemaker or Internal Cardioverter Defibrillator, details:________________

**□**3 Catheter ablation, details: _______________________________________

**□**4 Other, specify: ________________________________________________

***Record details of any present or past medications.***

1. {cva} **Stroke** **□**1 Yes **□**2 No **□**8 DK **□**9 Missing

- - 1. {cvayr} When was it diagnosed? _________ years ago
    2. {cvact} Diagnosis confirmed with a **CT scan**? **□**1 Yes **□**2 No **□**8 DK **□**9 Missing
    3. {cvadr} Name & address of doctor who made diagnosis?

___________________________________________________________________

- - 1. {cvahsp} Were you admitted to hospital? **□**1 Yes **□**2 No **□**8 DK **□**9 Missing
    2. {cvahspn} Which hospital? ______________________________________________________
    3. {cvahspt} For how long? _________ days
    4. {cvaaff} How did the stroke affect you? **□**1 Mild **□**2 Moderate **□**3 Severe
    5. {cvabod} Part of body affected:  **□**1 R Arm **□**2 L Arm **□**1 R Leg **□**2 L Leg

**□**3 Speech **□**4 Other:_____________

- - 1. {strkrec} How well have you recovered from the stroke? □□□% (100% is full recovery)
    2. {strkdur} How long did it take?________ months
    3. {strkmed} Do you take any medication as treatment for your stroke (see list)?

**□**1 Yes **□**2 No **□**8 DK **□**9 Missing

***Record details of any present or past medications.***

1. {tia} **Mini-stroke or TIA** **□**1 Yes **□**2 No **□**8 DK **□**9 Missing

(Stroke-like episodes with weakness in your face, fingers, hands, arms which last for short periods of time, transient loss of vision in one eye)

8-5.1 {tiayr} When was the first attack? ________ years ago

8-5.2 {tiaop} Did you ever have surgery to the brain or neck to correct or prevent a stroke? **□**1 Yes **□**2 No **□**8 DK **□**9 Missing

8-5.3 {tiahsp} Which hospital did you have this surgery at? ______________________________

8-5.4 {tiaopt} Surgery ________ years ago

8-5.5 {tiamed} Are you on any medication to prevent a stroke?

**□**1 Yes **□**2 No **□**8 DK **□**9 Missing

***Record details of any present or past medications.***

1. {ht} **High blood pressure** **□**1 Yes **□**2 No **□**8 DK **□**9 Missing

8-6.1 {htyr} When was it first diagnosed? ________ years ago

8-6.2 {htmed} Do you take medication to lower your blood pressure?

**□**1 Yes **□**2 No **□**8 DK **□**9 Missing

***Record details of any present or past medications.***

1. {chol} **High cholesterol** **□**1 Yes **□**2 No **□**8 DK **□**9 Missing

(On treatment or cholesterol > 5.5)

8-7.1 {cholyr} When was it first diagnosed? ________ years ago

8-7.2 {cholmed} Do you take medication to lower your cholesterol?

**□**1 Yes **□**2 No **□**8 DK **□**9 Missing

***Record details of any present or past medications.***

1. {peri} **Other vascular disease** **□**1 Yes **□**2 No **□**8 DK **□**9 Missing

(Peripheral vascular disease)

- - 1. {periyr} When was it first diagnosed? ________ years ago
    2. {peridia} Was the diagnosis confirmed with: **□**1 Ankle Brachial Pressure Index

**□**2 Ultrasound

**□**3 Angiography

**□**4 Computed Tomography (CT)

- - 1. {peridr} Name & address of doctor who made diagnosis?

___________________________________________________________________

8-8.4 {periyr} Treatment of vascular disease:

**□**1 Medications

**□**2 Angioplasty (PTA), details: ______________________________________

**□**3 Vascular surgery, details: _______________________________________

**□**4 Other, specify: ________________________________________________

***Record details of any present or past medications.***

1. {igt} **Pre-diabetes** or **impaired glucose tolerance**

**□**1 Yes **□**2 No **□**8 DK **□**9 Missing

8-9.1 {igtyr} When was it first diagnosed? __________ years ago

8-9.1 {igtdia} Was this diagnosed with: **□**1 Oral Glucose Tolerance Test

**□**2 Fasting Glucose Test

**□**3 Other, specify: ______________________________

8-9.1 {igtlife} Any lifestyle changes undertaken? **□**1 Yes **□**2 No

8-9.1 {igtmea} Measures taken: **□**1 Exercise from _______ to _______

**□**2 Modify diet from _______ to _______

**□**3 Weight reduction from _______ to _______

**□**4 Other, specify: ______________________________

1. {diab} **Diabetes**  **□**1 Yes **□**2 No **□**8 DK **□**9 Missing

(High sugar in the blood or urine)

8-10.1 {diabyr} When was it first diagnosed? __________ years ago

8-10.2 {diatyp} Type of diabetes: **□**1 Type 1 **□**2 Type 2 **□**3 Gestational Diabetes **□**4 Other: _______________**□**8 DK **□**9 Missing

In what year did you begin and finish each type of treatment?

(if currently on treatment put 7777 as year finished)

**Yes No DK Miss'g Started Finished**

8-10.3 {diadiet} Diet alone **□**1 **□**2 **□**8 **□**9 ______ ______

8-10.4 {diatab} Tablets **□**1 **□**2 **□**8 **□**9 ______ ______

8-10.5 {diains} Insulin **□**1 **□**2 **□**8 **□**9 ______ ______

8-10.6 {diabno} No treatment **□**1 **□**2 **□**8 **□**9 ______ ______

***Record details of any present or past medications.***

8-11 If **yes** to **pre-diabetes** or **diabetes**, complete the following questions.

8-11.1 {diabyr} Have you ever been told by a doctor you have **Polycystic Ovarian Syndrome**?

**□**1 Y **□**2 N  **□**3 N/A **□**8 DK **□**9 Missing

8-11.2 {dredyr} When were you first told? ________________ years ago

8-11.3 {dred} Have you ever been told by a doctor that you have eye disease or eye damage related to your diabetes (**Diabetic Retinopathy**)?

**□**1 Y **□**2 N **(go to 8-12)** **□**8 DK **□**9 Missing

8-11.4 {dredyr} When were you first told? ________________ years ago

8-11.5 {drlase} Have you ever had laser treatment for your diabetic eye disease?

**□**1 Y **□**2 N **(go to 8-12)** **□**8 DK **□**9 Missing

If **yes:**

|  |  | **Right** | **Left** |
| --- | --- | --- | --- |
| 8-11.6 | Which eye was treated? | {rdlas}  **Yes No DK Missing**  **□**1 **□**2 **□**8 **□**9 | {ldlas}  **Yes No DK Missing**  **□**1 **□**2 **□**8 **□**9 |
| 8-11.7 | How many years ago? | {rdlasyr}  __________ years ago | {ldlasyr}  __________ years ago |

8-11.8 {drlasdr} Which eye doctor performed the laser treatment?__________________________

__________­_______________________________________________________

1. {gout} **Gout** **□**1 Yes **□**2 No **□**8 DK **□**9 Missing

8-12.1 {goutyr} When was the first episode of gout? ________ years ago

8-12.2 {goutmed} Are you taking a medicine for gout?

**□**1 Yes **□**2 No **□**8 DK **□**9 Missing

***Record details of any present or past medications.***

1. {kiddx} **Kidney Disease** **□**1 Yes **□**2 No **□**8 DK **□**9 Missing

- - 1. {kiddx} When was this first diagnosed? __________ years ago
    2. {kidcaus} What was it caused by? ­­­­­­­­­­­_______________________________________________
    3. {kiddr} Name and address of treating kidney doctor?_______________________________

___________________________________________________________________

- - 1. {kidrx} What type of treatment are you receiving?

**□**1 None

**□**2 Dietary measures

**□**3 Medication ***Record details of any present or past medications.***

**□**4 Peritoneal dialysis: commenced _____ years ago at ___________ hospital

**□**5 Haemodialysis: commenced ________ years ago at ___________ hospital

**□**6 Kidney transplant: _________ years ago at __________________ hospital

**□**7 Other

**□**8 Missing

1. {livdx} **Liver disease** or **fatty liver** **□**1 Yes **□**2 No **□**8 DK **□**9 Missing

- - 1. {livdx} When was this first diagnosed? __________ years ago
    2. {livcaus} What was the diagnosis? ______________________________________________
    3. {livdr} Name and address of treating liver doctor? ________________________________

___________________________________________________________________

- - 1. {livrx} Treatment received? _______________________________________please specify

1. {thy} **Thyroid condition** **□**1 Yes **□**2 No **□**8 DK **□**9 Missing

8-15.1 {thyyr} When was it first diagnosed? ________ years ago

8-15.2 {thydx} What was your thyroid problem due to? ___________________________ (specify)

8-15.3 {thyact} At the time of diagnosis, was your thyroid problem:

**□**1 Underactive **□**2 Overactive **□**3 Normal activity

**□**8 DK **□**9 Missing

8-15.4 {thygoit} Did you have a goitre (enlarged thyroid gland)?

**□**1 Yes **□**2 No **□**8 DK **□**9 Missing

8-15.5 {thyrx} Indicate which treatment you received (can tick more than one box):

**□**1 Surgery: at _________ age________________________ type of surgery

**□**2 Radioactive iodine: from ________ years old for __________ months

**□**3 Thyroxine tablets: from ________ years old to ________ years old **□**6 Still taking

**□**4 Carbimazole / Neo-mercazole (anti-thyroid medication):

from ________ years old to ________ years old

**□**5 Propylthiouracil: from ________ years old to ________ years old

**□**7 No treatment

**□**8 DK **□**9 Missing

***Record details of any present or past medications.***

8-15.6 {thydr} Name and address of doctors treating your thyroid problem currently?

__________________________________________________________________

__________________________________________________________________

8-16 {migrain} Has a doctor ever told you that you suffer from **migraine**?

(severe headaches, usually on one side, at least 5 attacks during lifetime, lasting at least 4 hours and less than 3 days, may have changes in vision like zig-zag lines or blurring, often nausea or vomiting and you usually need to lie down with the lights off)

**□**1 Yes **□**2 No **□**8 DK **□**9 Missing

8-16.1 {mig1st} Age started: _____________ years old

8-16.2 {migstop} How old were you when you stopped getting migraines? _____________ years old

**□**8 Still getting them  **□**8 DK  **□**9 Missing

***Record details of any present or past medications.***

1. Have you had any other **serious illnesses** or **major operations**?

**□**1 Yes **□**2 No **□**8 DK **□**9 Missing

If **yes**, specify illness or operation and year:

{illop1} _____________________________________ │code___________ Year:_______________

{illop2} _____________________________________ │code___________ Year:_______________

{illop3} _____________________________________ │code___________ Year:_______________

{illop4} _____________________________________ │code___________ Year:_______________

{illop5} _____________________________________ │code___________ Year:_______________

{illop6} _____________________________________ │code___________ Year:_______________

{illop7} _____________________________________ │code___________ Year:_______________

{illop8} _____________________________________ │code___________ Year:_______________

**9.** **FAMILY HISTORY**

*Some cardiovascular diseases run in families, so I would like to ask you about any family history of cardiovascular disease or conditions which increase the risk of cardiovascular problems:*

*Direct relationship -* ***parents****,* ***children*** *or* ***brothers*** *and* ***sisters***

Have any been diagnosed with: **Yes No Don't Know Missing**

9-1 {famang} Angina **□**1 **□**2 **□**8 **□**9

9-2 {famami} Heart attack **□**1 **□**2 **□**8 **□**9

9-3 {famarr} Arrhythmia **□**1 **□**2 **□**8 **□**9

9-4 {famstr} Stroke (Mini-stroke or TIA) **□**1 **□**2 **□**8 **□**9

9-5 {famchol} High cholesterol **□**1 **□**2 **□**8 **□**9

9-6 {famhtn} High blood pressure **□**1 **□**2 **□**8 **□**9

9-7 {famhtn} Diabetes **□**1 **□**2 **□**8 **□**9

9-8 {famsd} Sudden death **□**1 **□**2 **□**8 **□**9

If **yes** to 9-1 to 9-8 of the above please complete the following table:

| **Relative** | **Code** | **Diagnosis** | **Age at onset** | **Code** |
| --- | --- | --- | --- | --- |
| *eg Brother* |  | *Angina* | *60* |  |
|  |  | *Diabetes* | *50* |  |
|  | 9-9a {fam1} |  |  | 9-9  {famhis1} |
|  |  |  |  | 9-10  {famhis2} |
|  | 9-11a {fam} |  |  | 9-11  {famhis3} |
|  |  |  |  | 9-12 {famhis4} |
|  | 9-13a {fam5} |  |  | 9-13 {famhis5} |
|  |  |  |  | 9-14 {famhis6} |
|  | 9-15a {fam7} |  |  | 9-15 {famhis7} |
|  |  |  |  | 9-16 {famhis8} |

**10. SLEEP QUESTIONS**

10-1 {snore} Have you ever been told that you snore in your sleep?

**□**1 Yes **□**2 No **□**8 DK **□**9 Missing

- 1. {choke} Have you ever been told that you choke or gasp in your sleep at night?

**□**1 Yes **□**2 No **□**8 DK **□**9 Missing

10-3 {stpbr} Have you ever been told that you stop breathing during your sleep?

**□**1 Yes **□**2 No **□**8 DK **□**9 Missing

10-4 {soml} Do you often feel sleepy during the day?

**□**1 Yes **□**2 No **(go to 10-5)** **□**8 DK **□**9 Missing

If **yes**, how likely are you to doze off or fall asleep in the following situations, in contrast to feeling just tired?

| **The Epworth Sleepiness Scale** | | | | | | |
| --- | --- | --- | --- | --- | --- | --- |
|  | **Situation** | **Chance of dozing** | | | | |
|  |  | **None** | **Slight** | **Mod.** | **High** | **NA** |
| 10-4.1  {slsit} | Sitting and reading | **□**0 | **□**1 | **□**2 | **□**3 | **□**7 |
| 10-4.2  {sltv} | Watching TV | **□**0 | **□**1 | **□**2 | **□**3 | **□**7 |
| 10-4.3  {slpub} | Sitting inactive in public place (eg theatre or meeting) | **□**0 | **□**1 | **□**2 | **□**3 | **□**7 |
| 10-4.4  {slcar} | As a passenger in a car for an hour without a break | **□**0 | **□**1 | **□**2 | **□**3 | **□**7 |
| 10-4.5  {slrest} | Lying down to rest in the afternoon when circumstances permit | **□**0 | **□**1 | **□**2 | **□**3 | **□**7 |
| 10-4.6  {sltalk} | Sitting and talking to someone | **□**0 | **□**1 | **□**2 | **□**3 | **□**7 |
| 10-4.7  {sllch} | Sitting quietly after lunch without alcohol | **□**0 | **□**1 | **□**2 | **□**3 | **□**7 |
| 10-4.8  {sltrf} | In a car, whilst stopped for a few minutes in traffic | **□**0 | **□**1 | **□**2 | **□**3 | **□**7 |

10-5 {slapn} Have you been told by a doctor that you have **sleep apnoea**?

**□**1 Yes **□**2 No **(go to 11-1)** **□**8 DK **□**9 Missing

10-6 {slapnyr} **If yes,** for how many years? _____________ years

10-7 {slapndx} Was the diagnosis confirmed on nocturnal polysomnogram / sleep study?

**□**1 Yes **□**2 No **□**8 DK **□**9 Missing

10-8 {slapnloc} **If yes**, was this study done at home or in a laboratory?

**□**1 Home **□**2 Lab **□**8 DK **□**9 Missing

10-9 {slapndr} Name and address of doctor who made diagnosis:____________________________

____________________________________________________________________

____________________________________________________________________

10-10 {slapnrx} What treatment have you received and year of treatment?

**□**1 Weight reduction, years ago: ________________

**□**2 Alcohol reduction, years ago: ________________

**□**3 Surgery, specify: ____________________________ years ago: __________

**□**4 Nasal CPAP/ Bi PAP, years ago: ________________

**□**5 Other, specify: _____________________________ years ago: __________

**□**8 DK

**□**9 Missing

**11. LIFESTYLE ASSESSMENT**

**Smoking**

11-1 {smokin} Have you ever smoked cigarettes, cigars or a pipe regularly? (regularly being at least weekly)

**□**1 Y **□**2 N **(go to 11-6)** **□**8 DK **□**9 Missing

If **yes**, which of the following have you ever regularly smoked:

**Yes No Age smoked from Average amount per week**

*E.g. age 15 years to 45 years*

11-2a {cighx} Cigarettes **□**1 **□**2 ____ age - ____ age ____ packs (20 per pack) │code_____

(Ready-made)

11-2b {cighx} Cigarettes **□**1 **□**2 ____ age - ____ age ____ packets of tobacco │code_____ (Roll-your-own)

11-2c {piphx} Pipe **□**1 **□**2 ____ age - ____ age ____ packs of pipe tobacco │code____

11-2d {cigar} Cigars **□**1 **□**2 ____ age - ____ age ____ cigars (number) │code_____

11-3a {stopsmk} Have you **given up** smoking? **□**1 Y **□**2 N **(go to 11-5)** **□**8 DK **□**9 Missing

11-3b {stopage} How old were you when you stopped smoking? _____________ years old

11-4 {pastcig} How much did you usually smoke per week just before you stopped?

_______ packs of manufactured cigarettes (20 per pack)

_______ packets of hand-rolled cigarettes

_______ cigars

_______ packets of pipe tobacco **(go to 11-6)**

11-5 {currcig} How much do you smoke per week **currently**?

_______ packs of manufactured cigarettes (20 per pack)

_______ packets of hand-rolled cigarettes

_______ cigars

_______ packets of pipe tobacco

11-6 {spsmk} Is your **husband/wife/partner**:

**□**1 A current smoker **(go to 11-7)** **□**4 Spouse deceased **(go to 11-12)** **□**2 An ex-smoker **(go to 11-9)**  **□**5 Divorced **(go to 11-12)** **□**3 A never smoker **(go to 11-18)** **□**6 No spouse/partner **(go to 11-18)**

**□**4 Don't know **(go to 11-18)**  **□**9 Missing

11-7 {spcig} **If spouse a current smoker:**

On average how much do you think your husband/wife/partner smokes per week?

_______ packs of manufactured cigs (20 per pack)

_______ packets of hand-rolled cigs

_______ cigars

_______ packets of pipe tobacco

11-8 {spsmyr} What year did he/she **start** smoking?

(if already smoking when married give year of marriage) _______ (year) **(go to 11-18)**

11-9 {spcig} **If spouse an ex-smoker:**

On average how much do you think your husband/wife/partner smoked **per week** just before he/she stopped?

_______ packs of manufactured cigs (20 per pack)

_______ packets of hand-rolled cigs

_______ cigars

_______ packets of pipe tobacco

11-10 {spsmyr} What year did he/she **start** smoking?

(if already smoking when married give year of marriage) _______ (year)

11-11 {spst} When did he/she **stop** smoking? Gave up in _______ (year) **(go to 11-18)**

11-12 {decsm} **If spouse deceased or divorced:**

At the time of death/divorce, was your husband/wife/partner

**□**1 A smoker **(go to 11-13)**

**□**2 An ex-smoker **(go to 11-15)** **□**8 Don't know **(go to 11-18)**

**□**3 A never smoker **(go to 11-18)**  **□**9 Missing **(go to 11-18)**

11-13 {decsm1} **If deceased/divorced spouse a smoker:**

At the time of death/divorce**,** on average how much do you think your husband/wife/partner smoked **per week**?

_______ packs of manufactured cigs (20 per pack)

_______ packets of hand-rolled cigs

_______ cigars

_______ packets of pipe tobacco

11-14 {decsm2} What year did he/she **start** smoking?

(if already smoking when married give year of marriage) _______ (year)

11-14a {decs2} In what year did he/she die? (**or** in what year did you separate?) _______ (year) **(go to 11-18)**

11-15 {decsm3} **If deceased/divorced spouse an ex-smoker:**

On average, how much do you think your husband/wife/partner smoked **per week** just before they stopped?

_______ packs of manufactured cigs (20 per pack)

_______ packets of hand-rolled cigs

_______ cigars

_______ packets of pipe tobacco

11-16 {decsm4} What year did he/she **start** smoking?

(if already smoking when married give year of marriage) _______ (year)

11-17 {decsm5} When did he/she **stop** smoking? Gave up in _______ (year)

**Alcohol**

11-18 {alc} How often do you have an alcoholic drink?

**□**1 Never **(go to 11-21)** **□**5 5-6 days a week

**□**2 < Once a week **□**6 Every day

**□**3 1-2 days a week **□**8 Don't know

**□**4 3-4 days a week **□**9 Missing

11-19 {alctype} What do you **mostly** drink?

**□**1 Light beer **□**5 Fortified wine

**□**2 Beer **□**6 Others

**□**3 Wine **□**8 Don't know

**□**4 Spirits **□**9 Missing

11-20 {alcnum} On days when you have a drink, how many standard drinks do you usually have?

(Standard drink = 10gm alcohol eg 250mL beer, 400mL light beer, 100mL wine)

**□**1 1-2  **□**5 13 or more

**□**2 3-4 **□**8 don't know

**□**3 5-8 **□**9 missing

**□**4 9-12

11-21 {alcpast} Has there ever been a time in your life when you regularly drank four or

more standard alcoholic drinks a day?

**□**1 Y **□**2 N **□**8 DK **□**9 Missing

**CLINICAL MEASUREMENTS / INVESTIGATIONS**

**DATE:** □□/□□/□□□□(dd/mm/yyyy)

**Section completed by:**________________________________________________

**12. CLINICAL MEASUREMENTS**

**Body Mass Index**

12-1.1 {height} **Height:** □•□□metres

12-1.2 {htrel} **□**1 Reliable

**□**2 Unreliable specify: _______________________________│code_________

**□**3 Not done specify: _______________________________│code_________

12-1.3 {wt} **Weight:** □□□kilograms

12-1.4 {wtrel} **□**1 Reliable

**□**2 Unreliable specify: _______________________________│code_________

**□**3 Not done specify: _______________________________│code_________

**Waist ratio**

12-2.1 {wst} **Waist:**□□□cm

12-2.2 {wstrel} **□**1 Reliable

**□**2 Unreliable specify: _______________________________│code_________

**□**3 Not done specify: _______________________________│code_________

# Please place BP cuff on arm, ensuring arm is supported at the level of the heart.

# Ask patient to uncross his / her legs.

**Heart Rate**

12-3.1 {hr} **Heart rate:**□□□beats per minute

12-3.2 {hrrel} **□**1 Reliable

**□**2 Unreliable specify: _______________________________│code_________

**□**3 Not done specify: _______________________________│code_________

**Blood pressure**

12-4.1 {armbp} I am now going to check your blood pressure. Indicate which arm used.

**□**1 R **□**2 L

**□**3 Not done specify: _______________________________

12-4.2 {caffbp} Have you had caffeine/ nicotine in last 4 hours? **□**1 Y **□**2 N **□**8 DK **□**9 Missing

12-4.3 {cuff} Cuff size: **□**1 Small **□**2 Adult **□**3 Large

12-4.4 {systbp} **Systolic BP:** □□□mmHg

12-4.5 {diasbp} **Diastolic BP:** □□□mmHg

12-4.6 {bprel} **□**1 Reliable

**□**2 Unreliable specify: _______________________________│code_________

**□**3 Not done specify: _______________________________│code_________

**SphygmoCor Px and Vx**

12-5 {sph}  **□**0 Not done

**□**1 Done, time of examination: _____________________________

12-5.1 **Aortic Blood Pressures** [systolic /diastolic (mean)]

{sphsbp} Systolic: □□□mmHg

{sphdbp} Diastolic: □□□mmHg

{sphmbp} Mean: □□□mmHg

12-5.2 {sphap} Augmented Pressure (AP): □□mmHg
12-5.3 {sphaix} Augmentation Index (AIx): □□%
12-5.4 {sphhr} Heart Rate: □□□bpm
12-5.5 {sphapp} Aortic Pulse Pressure: □□mmHg
12-5.6 {sphaix75} AIx@HR75: □□□%
12-5.7 {sphed} Ejection Duration: □□%
12-5.8 {sphsevr} Subendocardial viability ratio (SEVR): □□□%

Attach electrodes to patient’s right arm (white), left arm (black) and left leg (red)

12-5.9 {sphpwv} **Pulse Wave Velocity** ± the standard error

□□•□±□□•□m/s

12-5.10 {sphmea} Measured between (tick two points):

**□**1 **Carotid** **□**2 Femoral **□**3 Radial

12-5.11 {sphhrv} **Heart Rate Variability**

Valsalva Ratio □•□□

**Endothelial Function / Peripheral Arterial Tone – PAT2000**

12-6 {pat}  **□**0 Not done

**□**1 Done, time of examination: _____________________________

Ensure that the cuff (test) is placed on the patient’s non-dominant arm

12-6.1 {patarm} Occluded arm (test arm): **□**1 R **□**2 L

12-6.2 {patrhi} Reactive Hyperemia Index (RHI): □•□□
12-6.3 {pathr} Heart Rate: □□□bpm

**DATE:** □□/□□/□□□□(dd/mm/yyyy)

**Section completed by:** ________________________________________________

# 13. LABORATORY RESULTS

# Haematology

13-1 {haem}  **□**0 Not done

**□**1 Done, date / time of sample collection: ___________________________________

| **Parameter** | **Not Done** | **Value** | **Normals** | **Units** |
| --- | --- | --- | --- | --- |
| 13-1.1 {hhb} Haemoglobin | **□**0 |  | 130 - 180 | g/L |
| 13-1.2 {hwcc} White Cell Count | **□**0 |  | 3.7 – 9.5 | 10^9^/L |
| 13-1.3 {hpt} Platelets | **□**0 |  | 150 - 400 | 10^9^/L |
| 13-1.4 {hrcc} Red Cell Count | **□**0 |  | 4.3 – 5.7 | 10^12^/L |
| 13-1.5 {hhct} Haematocrit | **□**0 |  | 0.40 – 0.54 | % |
| 13-1.6 {hmcv} Mean Cell Volume | **□**0 |  | 82 - 98 | fL |

# Biochemistry

13-2 {bio} **□**0 Not done

**□**1 Done, date / time of sample collection: ___________________________________

| **Parameter** | **Not Done** | **Value** | | **Normals** | **Units** |
| --- | --- | --- | --- | --- | --- |
| 13-2.1 {biona} Sodium | **□**0 |  | | 135 - 145 | mmol/L |
| 13-2.2 {biok} Potassium | **□**0 |  | | 3.2 – 5.0 | mmol/L |
| 13-2.3 {biocl} Chloride | **□**0 |  | | 94 - 107 | mmol/L |
| 13-2.4 {bioco} CO_2_ | **□**0 |  | | 22 - 30 | mmol/L |
| 13-2.5 {biou} Urea | **□**0 |  | | 3.2 – 7.1 | mmol/L |
| 13-2.6 {bioc} Creatinine | **□**0 |  | | 55 – 105 | µmol/L |
| 13-2.7 {bioag} Anion Gap | **□**0 |  | | 12 - 20 | mmol/L |
| 13-2.8 {bioegfr} eGFR | **□**0 |  | | ≥ 90 | mL/min |
| 13-2.9 {biotb} Total Bilirubin | **□**0 |  | | < 21 | µmol/L |
| 13-2.10 {biotp} Protein | **□**0 |  | | 63 - 84 | g/L |
| 13-2.11 {bioal} Albumin | **□**0 |  | | 35 - 53 | g/L |
| 13-2.12 {biogl} Total Globulin | **□**0 |  | | 22 - 38 | g/L |
| 13-2.13 {bioalt} ALT | **□**0 |  | | < 47 | U/L |
| 13-2.14 {bioast} AST | **□**0 |  | | < 45 | U/L |
| 13-2.15 {bioggt} GGT | **□**0 |  | | < 43 | U/L |
| 13-2.16 {bioap} Alkaline Phosphatase | **□**0 |  | | 30 - 115 | U/L |
| **Parameter** | **Not Done** | **Value** | | **Normals** | **Units** |
| 13-2.17 {biock} Creatine Kinase (CK) | **□**0 | Admit | Peak | 55 - 170 | U/L |
| 13-2.18 {biott} TropT (cTnT) | **□**0 | Admit | Peak | 0.00 – 0.05 | µg/L |
| 13-2.19 {bioca} Calcium | **□**0 |  | | 2.13 – 2.63 | mmol/L |
| 13-2.20 {biocac} Calcium corrected | **□**0 |  | | 2.13 – 2.63 | mmol/L |
| 13-2.21 {biomg} Magnesium | **□**0 |  | | 0.65 – 1.05 | mmol/L |
| 13-2.22 {biopo} Phosphate | **□**0 |  | | 0.81 – 1.45 | mmol/L |
| 13-2.23 {biofg} Fasting Glucose | **□**0 |  | | 3.0 – 5.4 | mmol/L |

# Lipid Chemistry

13-3 {lip}  **□**0 Not done

**□**1 Done, Date / time of sample collection: ___________________________________

| **Parameter** | **Not Done** | **Value** | **Normals** | **Units** |
| --- | --- | --- | --- | --- |
| 13-3.1 {lipchol} Cholesterol | **□**0 |  | 2.5 – 5.5 | mmol/L |
| 13-3.2 {lipldl} LDL Cholesterol | **□**0 |  | 2.0 – 3.4 | mmol/L |
| 13-3.3 {liphdl} HDL Cholesterol | **□**0 |  | 0.9 – 2.0 | mmol/L |
| 13-3.4 {liptri} Triglyceride Fasting | **□**0 |  | 0.10 – 1.85 | mmol/L |

# Urine Chemistry

13-4 {uc} **□**0 Not done

**□**1 Done, date / time of sample collection: _____________________________________

| **Parameter** | **Not Done** | **Result** | | | | | |
| --- | --- | --- | --- | --- | --- | --- | --- |
| 13-4.1 {uc} Blood | **□**0 | Negative  **□**1 | Trace  **□**2 | +  **□**3 | ++  **□**4 | +++  **□**5 |  |
| 13-4.2 {ucph} pH | **□**0 | Acidic  **□**1 | Neutral  **□**2 | Alkaline  **□**3 |  |  |  |
| 13-4.3 {ucpro} Protein | **□**0 | Negative  **□**1 | Trace  **□**2 | +  **□**3 | ++  **□**4 | +++  **□**5 | ++++  **□**6 |
| 13-4.4 {ucturb} If positive,  Turbidity | **□**0 |  |  | +  **□**1 | ++  **□**2 | +++  **□**3 |  |
| 13-4.5 {ucglu} Glucose | **□**0 | Negative  **□**1 | Trace  **□**2 | +  **□**3 | ++  **□**4 | +++  **□**5 |  |
| 13-4.6 {ucket} If positive,  Ketones | **□**0 | Negative  **□**1 | Trace  **□**2 | +  **□**3 | ++  **□**4 | +++  **□**5 |  |

**DIAGNOSIS OF HEART DISEASE**

**DATE:** □□/□□/□□□□(dd/mm/yyyy)

**SECTION COMPLETED BY:** ______________________________________________

**14. CLINICAL INVESTIGATIONS**

# Electrocardiogram

14-1 {ecg} **□**0 Not done **□**9 Missing

**□**1 Done, date / time of examination: _________________________________________

**Score using the Minnesota Code Classification System for ECG Findings**

| **ECG Characteristics** | **Normal** | **Abnormal** | **ECG Finding(s) Code(s)** |
| --- | --- | --- | --- |
| 14-1.1 {ecgqs} **Q and QS Patterns** | **□**1 | **□**2 |  |
| 14-1.2 {ecgql} Anterolateral site (leads I, aVL, V6) | **□**1 | **□**2 |  |
| 14-1.3 {ecgqp} Posterior (inferior) site (leads II, III, aVF) | **□**1 | **□**2 |  |
| 14-1.4 {ecgqa} Anterior site (leads V1, V2, V3, V4, V5) | **□**1 | **□**2 |  |
| 14-1.5 {ecgax} **QRS Axis Deviation** | **□**1 | **□**2 |  |
| 14-1.6 {ecghr} **High Amplitude R Waves** | **□**1 | **□**2 |  |
| 14-1.7 {ecgj} **ST Junction and Segment Depression** | **□**1 | **□**2 |  |
| 14-1.8 {ecgjl} Anterolateral site (leads I, aVL, V6) | **□**1 | **□**2 |  |
| 14-1.9 {ecgjp} Posterior (inferior) site (leads II, III, aVF) | **□**1 | **□**2 |  |
| 14-1.10 {ecgja} Anterior site (leads V1, V2, V3, V4, V5) | **□**1 | **□**2 |  |
| 14-1.11 {ecgt} **T-Wave Items** | **□**1 | **□**2 |  |
| 14-1.12 {ecgtl} Anterolateral site (leads I, aVL, V6) | **□**1 | **□**2 |  |
| 14-1.13 {ecgtp} Posterior (inferior) site (leads II, III, aVF) | **□**1 | **□**2 |  |
| 14-1.14 {ecgta} Anterior site (leads V2, V3, V4, V5) | **□**1 | **□**2 |  |
| 14-1.15 {ecgav} **A-V Conduction Defect** | **□**1 | **□**2 |  |
| 14-1.16 {ecgvcd} **Ventricular Conduction Defect** | **□**1 | **□**2 |  |
| 14-1.17 {ecgarr} **Arrhythmias** | **□**1 | **□**2 |  |
| 14-1.18 {ecgst} **ST Segment Elevation** | **□**1 | **□**2 |  |
| 14-1.19 {ecgstl} Anterolateral site (leads I, aVL, V6) | **□**1 | **□**2 |  |
| 14-1.20 {ecgstp} Posterior (inferior) site (leads II, III, aVF) | **□**1 | **□**2 |  |
| 14-1.21 {ecgsta} Anterior site (leads V1, V2, V3, V4,V5) | **□**1 | **□**2 |  |
| 14-1.22 {ecgmi} **Miscellaneous Items** | **□**1 | **□**2 |  |

**Diagnostic Angiography**

14-2 {da} **□**0 Not done **□**9 Missing

**□**1 Done, date / time of examination: _________________________________________

14-2.1 **Baseline Aortic Pressures** **Post Angio Aortic Pressures**

{basbp} Systolic: □□□mmHg {basbp} Systolic: □□□mmHg

{badbp} Diastolic: □□□mmHg {badbp} Diastolic: □□□mmHg

{bambp} Mean: □□□mmHg {bambp} Mean: □□□mmHg

**Vessel state**

0. Not present

1. Normal

2. Minor irregularities

3. Local narrowing <50%

4. Local narrowing 50-75%

5. Local narrowing >75%

6. Complete occlusion

7. Diffuse disease

|  | **1** | **2** | **3** | **4** | **5** | **6** | **7** |
| --- | --- | --- | --- | --- | --- | --- | --- |
| **Right** |  |  |  |  |  |  |  |
|  | **8** | **9** | **10** | **11** | **12** | **13** | **14** |
| **Left Main and LAD** |  |  |  |  |  |  |  |
|  | **15** | **16** | **17** | **18** | **19** | **20** |  |
| **Left Intermediate & Cx** |  |  |  |  |  |  |

14-2.2 {dag} **Grafts** ****1 Yes (specify below) ****2 No

**Graft state**

0. Not present

1. Normal

2. Minor irregularities

3. Local narrowing <50%

4. Local narrowing 50-75%

5. Local narrowing >75%

6. Complete occlusion

7. Diffuse disease

| **Graft** | **Graft State** |
| --- | --- |
| {g1} |  |
| {g2} |  |
| {g3} |  |
|  |  |

14-2.3 {dac} Comments:

__________________________________________________________________

__________________________________________________________________

14-2.4 {dalv} Left ventriculography performed **□**1 Yes **□**2 No

If **yes**, complete table:

|  | **Observation** | **Severity** | **Comment** |
| --- | --- | --- | --- |
| {dalvs} Left ventricle | **□**1 Normal  **□**2 Dilated |  |  |
| {dalvc} Left ventricle contractility | **□**1 Normal  **□**2 Impaired |  |  |

**Severity**  1. Mild 2. Moderate 3. Severe 8. Unknown

14-2.4 {dadia} **Diagnosis of coronary artery disease**

**□**1 Single vessel disease (1VD) (> 50% stenosis) **□**5 Minor disease (20% to 50% stenosis)

**□**2 Double vessel disease (2VD) (> 50% stenosis) **□**6 Normal (< 20% stenosis)

**□**3 Triple vessel disease (3VD) (> 50% stenosis) **□**7 Bifurcation

**□**4 Left main disease (> 50% stenosis) **□**8 Trifurcation

**New York Heart Association Functional Classification of Heart Failure**

14-3 {nyha} Classification of heart failure:

**□**1 **Class I:** No symptoms and no limitation in ordinary physical activity.

**□**2 **Class II:** Mild symptoms and slight limitation during ordinary activity. Comfortable at rest.

**□**3 **Class III:** Marked limitation in activity due to symptoms, even during less-than-ordinary activity. Comfortable only at rest.

**□**4 **Class IV:** Severe limitations. Experiences symptoms even while at rest.

**□**9 Not done, specify: _______________________________________________

# Chest X-Ray (CXR)

14-4 {cx} **□**0 Not done **□**9 Missing

**□**1 Done, date / time of examination: ______________________________________

# 14-4.1 {cxmeg} Cardiomegaly □1 Yes □2 No □3 Not requested

14-4.2 {cxpo} **Pulmonary odema** **□**1 Yes **□**2 No **□**3 Not requested

14-4.3 {cxcom} Comments: _____________________________________________________________________

_____________________________________________________________________

# MIBI Stress Test

14-5 {mib} **□**0 Not done **□**9 Missing

**□**1 Done, date / time of examination: __________________________________

**At Rest Test Conditions**

14-5.1 {mirhr} HR: □□□ beats per minute {mithr} HR: □□□ beats per minute

14-5.2 {mirbp} BP: □□□ /□□□mmHg {mitbp} BP: □□□ /□□□mmHg

14-5.3 {mirbp} Left ventricle dilatation: **□**1 Not present

**□**2 Present, severity: **□**1 Mild **□**2 Moderate

**□**3 Severe **□**4 Unknown

14-5.4 {mirbp} LV end-diastolic volume: □□□mL

14-5.5 {mirbp} LV Ejection Fraction: □□%

14-5.6 {mipdr} Presence of perfusion defect region(s):

**□**1 Not present **□**2 Present

If **present**, complete table:

|  | **Location** | **Reversibility** | **Size** | **Comment / Intensity** |
| --- | --- | --- | --- | --- |
| Region 1 |  | **□**1 Fully  **□**2 Partial  **□**3 Nil - fixed | **□**1 Small  **□**2 Moderate  **□**3 Large |  |
| Region 2 |  | **□**1 Fully  **□**2 Partial  **□**3 Nil - fixed | **□**1 Small  **□**2 Moderate  **□**3 Large |  |
|  | **Location** | **Reversibility** | **Size** | **Comment / Intensity** |
| Region 3 |  | **□**1 Fully  **□**2 Partial  **□**3 Nil - fixed | **□**1 Small  **□**2 Moderate  **□**3 Large |  |
| Region 4 |  | **□**1 Fully  **□**2 Partial  **□**3 Nil - fixed | **□**1 Small  **□**2 Moderate  **□**3 Large |  |

**Exercise Stress Test (EST)**

14-6 {est} **□**0 Not done **□**9 Missing

**□**1 Done, date / time of examination: __________________________________

**At Rest Maximum - Test Conditions**

14-6.1 {strhr} HR: □□□ beats per minute {stthr} HR: □□□ beats per minute

14-6.2 {strbp} BP: □□□ /□□□mmHg {sttbp} BP: □□□ /□□□mmHg

14-6.3 {strbp} Maximum predicted HR: □□□ beats per minute

14-6.4 {estend} Reason for termination

**□**1 Chest pain **□**4 Pain elsewhere

**□**2 Shortness of breath **□**5 Other, specify: _____________________

**□**3 Pre-syncope / syncope

14-6.5 {estarr} Stress ECG - arrhythmias detected:

**□**1 None (sinus rhythm) **□**4 Ventricular tachycardia (VT)

**□**2 Atrial fibrillation / flutter (AF) **□**5 Supraventricular tachycardia (SVT)

**□**3 Ventricular ectopic beats (VEBs) **□**6 Other, specify: ______________________

14-6.6 {estarr} ST segment depression: **□**1 Yes **□**2 No

If **yes**, complete table:

|  | **Location** | **Lead(s)** | **Max Depression (mm)** | **Slope** |
| --- | --- | --- | --- | --- |
| Region 1 |  |  |  |  |
| Region 2 |  |  |  |  |
| Region 3 |  |  |  |  |
| Region 4 |  |  |  |  |

**Location OR Lead(s) Slope**

Inferior I, II, III 1. Upsloping

Lateral V1, V2, V3 2. Planar

Anterior V4, V5, V6 3. Downsloping

Posterior aVL, aVR, aVF 8. Unknown

# Transthoracic Echocardiogram (TTE)

14-7 {tte} **□**0 Not done **□**9 Missing

**□**1 Done, date / time of examination: __________________________________

| **Parameter** | **Normals** | **Not Done** | **Value** | **Units** |
| --- | --- | --- | --- | --- |
| 14-7.1 {ttelvd} LV Internal Ø Diastole | 35 - 56 | **□**0 |  | mm |
| 14-7.2 {ttelvs} LV Internal Ø Systole | 20 - 40 | **□**0 |  | mm |
| 14-7.3 {ttela} Left Atrium | 24 – 40 | **□**0 |  | mm |
| 14-7.4 {tteev} E Velocity | | **□**0 |  | m/sec |
| 14-7.5 {tteav} A Velocity | | **□**0 |  | m/sec |
| 14-7.6 {ttepvs} Pulmonary vein systolic velocity | | **□**0 |  | m/sec |
| 14-7.7 {ttepvd} Pulmonary vein diastolic velocity | | **□**0 |  | m/sec |
| 14-7.8 {tterv} RVSP | | **□**0 |  | mmHg |

| **Parameter** | **Not Done** | **Score** |
| --- | --- | --- |
| 14-7.9 {ttelvv} Left ventricle volume | **□**0 |  |
| 14-7.10 {ttelvs} Left ventricle systolic function | **□**0 |  |
| 14-7.11 {ttervv} Right ventricle volume | **□**0 |  |
| 14-7.12 {ttervs} Right ventricle systolic function | **□**0 |  |
| 14-7.13 {ttelav} Left atrium volume | **□**0 |  |
| 14-7.14 {tterav} Right atrium volume | **□**0 |  |
| 14-7.15 {tteph} Pulmonary hypertension | **□**0 |  |

**Score**

0. Normal

1. Mild

2. Mild to moderate

3. Moderate

4. Moderate to severe

5. Severe

14-7.16 {ttecom} Comments:

____________________________________________________________________

____________________________________________________________________

# Gated Heart Pool Scan

14-8 {ghps} **□**0 Not done **□**9 Missing

**□**1 Done, date / time of examination: __________________________________

|  | **Observation** | **Severity** | **Comment / Kinesis and Region** |
| --- | --- | --- | --- |
| 14-8.1 {ghlvs} Right ventricle | **□**1 Normal  **□**2 Dilated |  |  |
| 14-8.2 {ghlvs} Right ventricle contractility | **□**1 Normal  **□**2 Impaired |  |  |
| 14-8.3 {ghlvs} Left ventricle | **□**1 Normal  **□**2 Dilated |  |  |
| 14-8.4 {ghlvc} Left ventricle  contractility | **□**1 Normal  **□**2 Impaired |  |  |

**Severity**  **Kinesis** **Region**

14-8.5 {ghlvc} LV Ejection Fraction: □□%

1. Mild 2. Hypokinesia Use segment

2. Moderate 3. Akinesia numbers

3. Severe 4. Dyskinesia (ACC)

5. Aneurysmal

# Treatments / Interventions

14-9 {pts} Primary treatment strategy:

**□**1 Percutaneous transluminal coronary angioplasty (PTCA) and stent

**□**2 Direct stenting

**□**3 Percutaneous transluminal coronary angioplasty (PTCA) only

**□**4 Immediate coronary artery bypass grafting (CABG)

**□**5 Delayed coronary artery bypass grafting (CABG)

**□**6 Elective coronary artery bypass grafting (CABG)

**□**7 Thrombolysis

**□**8 Medical management

**□**9 Nil

**□**10 PTCA then immediate CABG

Comments:

___________________________________________________________________

# ___________________________________________________________________

# VISUAL FUNCTION AND EYE CONDITIONS

**15. VISUAL FUNCTION AND CHANGE IN VISUAL FUNCTION**

**Use of Glasses**

- 1. {glass} Do you wear **glasses** of any kind?

**□**1 Yes **□**2 No **(go to 15-7)** **□**8 DK **□**9 Missing

- 1. {typgls} If **yes**, are they:

**□**1 Single vision distance glasses only

**□**2 Single vision reading glasses only

**□**3 Separate reading and distance glasses

**□**4 Bifocals **□**8 Don't know

**□**5 Multifocals **□**9 Missing

15-3 {agegls} How old were you when you first needed to wear glasses to see clearly in the

**distance**?

_________ years old **□**1 Don’t wear distance glasses **□**8 DK **□**9 Missing

15-4 {presby} How old were you when you first needed **reading glasses, bifocals or multifocals**?

_________ years old **□**1 Don’t wear reading glasses **□**8 DK **□**9 Missing

- 1. {timegls} How long have you had your current glasses?

Glasses are _________ years old **□**8 DK **□**9 Missing

- 1. {rdnews} Can you read the ordinary print in the newspaper reasonably well, with or without

glasses? **□**1 Yes **(go to 15-9) □**2 No **□**8 DK **□**9 Missing

- 1. {lastrd} **If no**, when were you last able to do this? _________ years ago **□**8 DK **□**9 Missing

**Eye Symptoms**

- 1. {Vworse} Are you aware of a **deterioration of vision** in one or both eyes?

**□**1 Yes, R eye **(go to 15-9)** **□**4 No **(go to 16-1)**

**□**2 Yes, L eye **(go to 15-10)** **□**8 Don't know

**□**3 Yes, both eyes **□**9 Missing

- 1. {Rworse} When did your right eye worsen? ___________ months ago
  2. {Lworse} When did your left eye worsen? ___________ months ago

**16. EYE DISEASE**

**Cataract**

1. {cat} Have you ever been told by a doctor that you have a **cataract**?

**□**1 Y **□**2 N **(go to 16-9)** **□**8 DK **□**9 Missing

1. {catrl} If **yes**, in which eye? **□**1 Right eye **□**2 Left eye

| **Cataract** | **Right Eye** | **Left Eye** |
| --- | --- | --- |
| 1. In what year were you first told? | {rcatyr} | {lcatyr} |
| 1. Have you had an operation for cataract? | {rcatop}  **Yes No DK Missing**  **□**1 **□**2 **□**8 **□**9 | {lcatop}  **Yes No DK Missing**  **□**1 **□**2 **□**8 **□**9 |
| 1. If **yes**, in what year? | {rcatop} | {lcatop} |
| 1. Have you had YAG laser to improve your vision after cataract surgery? | {rcatYAG}  **Yes No DK Missing**  **□**1 **□**2 **□**8 **□**9 | {lcatYAG}  **Yes No DK Missing**  **□**1 **□**2 **□**8 **□**9 |
| 1. If **yes**, in what year? | {rcatYGy} | {lcatYGy} |
| 1. Eye doctor treating your cataract? | {rcatdr} | {rcatdr} |

**Macular Degeneration**

1. {AMD} Have you ever been told by a doctor that you have **macular degeneration**?

(hardening of the arteries at the back of the eye or retinal degeneration)

**□**1 Y **□**2 N **(go to 16-15)** **□**8 DK **□**9 Missing

1. {AMDrl} If **yes**, in which eye? **□**1 Right eye **□**2 Left eye

| **Macular degeneration** | **Right Eye** | **Left Eye** |
| --- | --- | --- |
| 1. In what year were you first told? | {rAMDyr} | {lAMDyr} |
| 1. Have you had laser treatment for macular   degeneration? | {rAMDlas}  **Yes No DK Missing**  **□**1 **□**2 **□**8 **□**9 | {lAMDlas}  **Yes No DK Missing**  **□**1 **□**2 **□**8 **□**9 |
| 1. If **yes**, in what year did you first have laser treatment? | {rlasy} | {llasy} |
| 1. Which eye doctor performed the laser treatment? | {rlasdr} | {llasdr} |

Glaucoma

1. {glaucom} Have you ever been told by a doctor that you have **glaucoma?**

**□**1 Y **□**2 N **(go to 16-23)** **□**8 DK **□**9 Missing

1. {glauyr} If **yes**, in what year were you first told? _____________(year)
2. {glauRx} Have you used eyedrops or other medications for glaucoma?

**□**1 Y **□**2 N **(go to 16-21)** **□**8 DK **□**9 Missing

1. {glRxyr} If **yes**, in what year did you start using these medications? _____________(year)
2. {glRxyrs} For how many years? ____________ years
3. {glaucdr} Which eye doctor **first** put you on treatment for glaucoma? ___________________

__________________________________________________________________

1. {glausur} Have you had an operation or laser treatment for glaucoma?

**□**1 Y  **□**2 N **(go to 16-23)** **□**8 DK **□**9 Missing

1. {glsuryr} If **yes**, in what year? _____________(year)

**Other Eye Conditions**

1. {retina} Have you ever been told that you have a **problem in the retina** or the **'back of the**

**eye'** eg retinal detachment, vessel blockage or bleeding?

**□**1 Y **□**2 N **(go to 16-25)** **□**8 DK **□**9 Missing

1. {retdet} If **yes**, specify (which eye, condition and year):

_________________________________________________________│code______

1. Any **other eye problems or surgery** I haven't asked you about?

{other1} _________________________________________________________│code______

{other2} _________________________________________________________│code______

**17. DISABILITY**

**General -** To be completed by the **examiner**

**Does the participant:**  **Yes No Don't know Missing**

22-1 {D1} Have a hearing impairment **□**1 **□**2 **□**8 **□**9

22-2 {D2} Have walking difficulties **□**1 **□**2 **□**8 **□**9

22-3 {D3} Use a cane / crutches / walker **□**1 **□**2 **□**8 **□**9

22-4 {D4} Use a wheel chair **□**1 **□**2 **□**8 **□**9

22-5 {D5} Have SOB / cough continuously **□**1 **□**2 **□**8 **□**9

22-6 {D6} Have a language problem **□**1 **□**2 **□**8 **□**9

22-7 {D7} Have a speech but not a language problem **□**1 **□**2 **□**8 **□**9

22-8 {D8} Appear demented **□**1 **□**2 **□**8 **□**9

22-9 {D9} **Who mainly answered the questionnaire?**

**□**1 Participant **□**4 Sibling **□**7 Other specify: _________ |code_____

**□**2 Spouse **□**5 Other relative **□**8 DK

**□**3 Son/daughter **□**6 Friend **□**9 Missing

22-10 {D10} The overall quality of the interview was:

**□**1 Reliable **□**2 Unreliable **□**8 DK **□**9 Missing

**18. RETINAL PHOTOGRAPHY**

18.1 {whenrp} When was retinal photography performed?

**□**1 **Before** Coronary Catheter Laboratory

**□**2 **After** Coronary Catheter Laboratory

**□**3 **Before and after** Coronary Catheter Laboratory

**□**4 Not done, reason:

_______________________________________________________

**Before Cath Lab**

Date of photography: □□/□□/□□□□(dd/mm/yyyy)

Photography performed by: ___________________________________________________

Location: _________________________________________________________________

DILATION: Tropicamide 1% and Phenylephrine 10% in both eyes

**□**1 Drops instilled, time: □□:□□

**□**2 Drops not instilled, reason: _________________________________________________

|  | **Camera** | **Both** | **Right only** | **Left only** | **None** | **Reason for inability to take photograph** |
| --- | --- | --- | --- | --- | --- | --- |
| {befmyd} | Canon (Fundus) | **□**1 | **□**2 | **□**3 | **□**4 |  |
| {befnon} | Canon (Non-mydriatic) | **□**1 | **□**2 | **□**3 | **□**4 |  |

**After Cath Lab**

Date of photography: □□/□□/□□□□(dd/mm/yyyy)

Photography performed by: ___________________________________________________

Location: _________________________________________________________________

DILATION: Tropicamide 1% and Phenylephrine 10% in both eyes

**□**1 Drops instilled, time: □□:□□

**□**2 Drops not instilled, reason: _________________________________________________

|  | **Camera** | **Both** | **Right only** | **Left only** | **None** | **Reason for inability to take photograph** |
| --- | --- | --- | --- | --- | --- | --- |
| {aftmyd} | Canon (Fundus) | **□**1 | **□**2 | **□**3 | **□**4 |  |
| {aftnon} | Canon (Non-mydriatic) | **□**1 | **□**2 | **□**3 | **□**4 |  |

**19. VISUAL ACUITY AND SUBJECTIVE REFRACTION**

E-0 {va} **□**0 Not done

*I am now going to test your vision with your glasses for distance vision, if you wear them.*

**Logmar visual acuity score or E – equivalent**

*Measure at 2.4 metres (8 ft) with best distance correction; if unable to see any letters, then try at one metre.*

1. What distance was chart read? {Rrddist} **R** **□**1 2.4m {Lrddist} **L** **□**1 2.4m **□**2 1.2m **□**2 1.2m
2. Current distance glasses worn? {currdrx} **□**1 Yes **□**2 No

**Visual Acuity**

*2.4 m* Right eye no. correct Left eye no. correct

*6/60*  H V Z D S ____ H V Z D S ____ 5

*6/48* N C V K D ____ N C V K D ____ 10

*6/36* C Z S H N ____ C Z S H N ____ 15

*6/30* O N V S R ____ O N V S R ____ 20

*6/24* K D N R O ____ K D N R O ____ 25

*6/19* Z K C S V ____ Z K C S V ____ 30

*6/15* D V O H C ____ D V O H C ____ 35

*6/12* O H V C K ____ O H V C K ____ 40

*6/9.5* H Z C K O ____ H Z C K O ____ 45

*6/7.5* N C K H D ____ N C K H D ____ 50

*6/6* Z H C S R ____ Z H C S R ____ 55

*6/4.8*  S Z R D N ____ S Z R D N ____ 60

*6/3.8* H C D R O ____ H C D R O ____ 65

*6/3.0* R D O S N ____ R D O S N ____ 70

**Right eye** **Left eye**

1. {RmarVA} Logmar VA _________ {LmarVA} Logmar VA _________
2. {RPH} Pinhole _________ {LPH} Pinhole _________

**If vision < 6/60**

1. {RpoorVA} **Right** {LpoorVA} **Left**

**□**1 CF **□**1 CF

**□**2 HM **□**2 HM

**□**3 PL **□**3 PL

**□**4 NPL **□**4 NPL

**Logmar VA modified Sheridan-Gardiner** (only if unable to read chart)

1. {RSheGar} **R** _________ {LSheGar} **L**  _________
2. {amblyo} **If one eye weaker (2 line difference) ask:**

Has your Right/Left eye always been weaker?

**□**1 Right eye – yes **□**8 DK

**□**2 Left eye – yes **□**9 Missing

**□**3 No

1. {visdis} **If visual disability, eg field defect or severe visual loss (< 6/60) in both eyes, ask:**

Have you sought help from:

**□**1 Low vision clinic

**□**2 Royal Blind Society

**□**3 Guide dogs

**□**4 Other agency

specify: _________________ |code ______

1. {demenVA} Did mental disability or dementia prevent measurement of VA?

**□**1 Yes **□**2 No **□**8 DK **□**9 Missing

**Visual Acuity with best subjective refraction**

*2.4 m* Right eye no. correct Left eye no. correct

*6/60* H V Z D S ____ H V Z D S ____ 5

*6/48*  N C V K D ____ N C V K D ____ 10

*6/36* C Z S H N ____ C Z S H N ____ 15

*6/30* O N V S R ____ O N V S R ____ 20

*6/24* K D N R O ____ K D N R O ____ 25

*6/19* Z K C S V ____ Z K C S V ____ 30

*6/15* D V O H C ____ D V O H C ____ 35

*6/12* O H V C K ____ O H V C K ____ 40

*6/9.5* H Z C K O ____ H Z C K O ____ 45

*6/7.5* N C K H D ____ N C K H D ____ 50

*6/6* Z H C S R ____ Z H C S R ____ 55

*6/4.8*  S Z R D N ____ S Z R D N ____ 60

*6/3.8* H C D R O ____ H C D R O ____ 65

*6/3.0* R D O S N ____ R D O S N ____ 70

1. {RsubjVA} Logmar VA **Right** _________ {LsubjVA} Logmar VA **Left** _________
