# Supplementary material for: Associations between retinal arteriolar and venular calibre with the prevalence of impaired fasting glucose and diabetes mellitus: A cross-sectional study
Source: PLoS One. 2018 May 3;13(5):e0189627. doi: 10.1371/journal.pone.0189627 (PMC5933737; doi:10.1371/journal.pone.0189627)
Supplement: S4 File — Table A. Baseline characteristics. Table B. Cross-sectional association between adjusted retinal vessel calibre and impaired fasting glucose (IFG). Table C. Relationship between retinal venular calibre and diabetes, stratified by Gemini / Extent score, according to median. (DOCX) [file pone.0189627.s004.docx]

**Table A. Baseline characteristics**

| **Characteristics** | **Included participants (n=748)** | **Excluded participants (n=932)** | **P-value** |
| --- | --- | --- | --- |
| Age | 60.71±11.62 | 62.23±11.58 | 0.008 |
| Male | 585 (78.2%) | 684 (73.4%) | 0.022 |
| BMI | 29.47±5.58 | 29.58±5.66 | 0.71 |
| Smoking | 250 (33.65%) | 197 (21.16%) | <0.001 |
| History of diabetes | 270 (36.1%) | 295 (31.75%) | 0.06 |
| History of hypertension | 465 (62.3%) | 731 (78.9%) | <0.001 |
| Alcohol consumption (rarely+often) | 545 | 642 | 0.02 |
| HDL | 1.03±0.29 | 1.04±0.31 | 0.38 |
| Cholesterol | 4.61±1.11 | 4.56±1.31 | 0.49 |
| Statins | 108 (14.4%) | 183 (19.6%) | 0.005 |
| Retinal arteriolar calibre (µm) | 148.29±15.08 | 146.59±14.03 | 0.07 |
| Retinal venular calibre (µm) | 224.74±22.45 | 221.81±21.78 | 0.04 |

BMI, body mass index; HDL, high density lipoproteins

**Table B.** Cross-sectional association between adjusted retinal vessel calibre and impaired fasting glucose (IFG)

| **Retinal vessel calibre** | **Men (n=443)** | | | | **Women (n=114)** | | | |
| --- | --- | --- | --- | --- | --- | --- | --- | --- |
|  | **Age-adjusted OR** | **P-value** | **Multivariable adjusted OR** | **P-value** | **Age-adjusted OR** | **P-value** | **Multivariable adjusted OR** | **P-value** |
| Adjusted retinal arteriolar calibre (µm) |  |  |  |  |  |  |  |  |
| 1^st^ tertile | 1.40 (0.77-2.56) | 0.27 | 1.24 (0.59-2.59) | 0.57 | 0.76 (0.20-2.89) | 0.69 | 0.43 (0.05-3.71) | 0.44 |
| 2^nd^ tertile | 1.41 (0.76-2.62) | 0.29 | 1.05 (0.48-2.33) | 0.90 | 0.4842 (0.13-1.79) | 0.28 | 0.41 (0.07-2.56) | 0.34 |
| 3^rd^ tertile | 1.0 (reference) | - | 1.0 (reference) | - | 1.0 (reference) | - | 1.0 (reference) | - |
| Adjusted retinal venular calibre (µm) |  |  |  |  |  |  |  |  |
| 1^st^ tertile | 1.0 (reference) | - | 1.0 (reference) | - | 1.0 (reference) | - | 1.0 (reference) | - |
| 2^nd^ tertile | 1.26 (0.70-2.30) | 0.45 | 1.55 (0.74-3.261) | 0.25 | 0.67 (0.17-2.60) | 0.57 | 0.57 (0.09-3.47) | 0.54 |
| 3^rd^ tertile | 1.30 (0.70-2.40) | 0.41 | 1.42 (0.67-3.04) | 0.36 | 0.54 (0.14-2.07) | 0.37 | 0.30 (0.04-2.32) | 0.25 |

*Multivariate analysis: age, BMI, alcohol, smoker, hypertension, history of diabetes, cholesterol level, HDL level, prior statin use.

OR, odds ratio; CI, confidence interval; SD, standard deviation

**Table C.** Relationship between retinal venular calibre and diabetes, stratified by Gemini / Extent score, according to median

|  | **Adjusted Retinal venular calibre per SD increase** | | | |
| --- | --- | --- | --- | --- |
|  | **Age-sex adjusted OR (95% CI)** | **P-value** | **Multivariable adjusted OR (95% CI)** | **P-value** |
| By Gensini score  ≤ median (34)  > median (34) | 1.23 (0.99-1.72)  1.17 (0.92-1.49) | 0.22  0.21 | 1.07 (0.70-1.64)  1.12 (0.84-1.48) | 0.75  0.45 |
| By Extent score  ≤ median (36.9)  > median (36.9) | 1.69 (1.15-2.48)  1.01 (0.77-1.34) | 0.008  0.92 | 1.50 (0.96-2.35)  0.94 (0.66-1.32) | 0.08  0.70 |

*Multivariable analysis: age, BMI, alcohol, smoker, hypertension, history of diabetes, cholesterol level, HDL level, prior statin use.

OR, odds ratio; CI, confidence interval; SD, standard deviation
